# Continuous positive airway pressure is unsafe for radiofrequency ablation of lung cancer under sedation: a randomised controlled trial

**DOI:** 10.1186/s13244-024-01721-9

**Published:** 2024-06-20

**Authors:** Enrique Carrero-Cardenal, Ivan Vollmer-Torrubiano, Marta Torres-López, Gloria Martín-Barrera, Georgina Casanovas-Mateu, Francisco-Javier Tercero-Machin, Alfredo Paez-Carpio, Neus Fábregas-Julià, Ricard Valero-Castell

**Affiliations:** 1 Department of Anaesthesiology, Hospital Clínic Barcelona, Universitat de Barcelona, Barcelona, Spain; 2 Institute for Biomedical Research August Pi i Sunyer (IDIBAPS), Barcelona, Spain; 3 Department of Radiology, Hospital Clínic Barcelona, Universitat de Barcelona, Barcelona, Spain; 4 Centre for Biomedical Research in the Respiratory Diseases Network (CIBERES), Instituto de Salud Carlos III, Madrid, Spain; 5 Surgical Area Nursing Department, Hospital Clínic Barcelona, Universitat de Barcelona, Barcelona, Spain; 6 Clinical Quality and Safety Directorate, Surgical Area, Hospital Clínic Barcelona, Universitat de Barcelona, Barcelona, Spain; 7 Centre for Biomedical Research Network on Mental Health (CIBERSAM), Instituto de Salud Carlos III, Madrid, Spain

**Keywords:** Conscious sedation, Continuous positive airway pressure, Lung cancer, Patient safety, Radiofrequency ablation

## Abstract

**Objective:**

To evaluate the safety of a minimum continuous positive airway pressure of 4 cmH_2_O (CPAP + 4) during computed tomography (CT)-guided radiofrequency ablation (RFA) for lung malignancies under procedural sedation and analgesia (PSA).

**Methods:**

This was a prospective, randomised, single-blind, parallel-group, placebo-controlled trial with an open-label medical device conducted at a single tertiary university hospital in Barcelona, Spain. Forty-six patients over 18 years of age scheduled for CT-guided RFA of a malignant pulmonary tumour under PSA were randomised to receive either CPAP + 4 or a modified mask for placebo CPAP (Sham-CPAP). Exclusion criteria included contraindications for RFA, refusal to participate, inability to understand the procedure or tolerate the CPAP test, lung biopsy just prior to RFA, intercurrent diseases, or previous randomisation for additional pulmonary RFA. Primary outcomes were the percentage of patients reporting at least one serious adverse event (SAE), classification for complications from the Cardiovascular and Interventional Radiological Society of Europe (CIRSE), and Clavien-Dindo classifications for complications, hospital stay, and readmissions. Secondary outcomes included adverse events (AEs), respiratory parameters, airway management, and the local radiological efficacy of pulmonary ablation.

**Results:**

CPAP + 4 prolonged hospital stay (1.5 ± 1.1 vs. 1.0 ± 0 inpatient nights, *p* = 0.022) and increased the risk of AE post-RFA (odds ratio (95% CI): 4.250 (1.234 to 14.637), *p* = 0.021 with more pneumothorax cases (*n* = 5/22, 22.7% vs. *n* = 0/24, 0%, *p* = 0.019). Per-protocol analysis revealed more SAEs and CIRSE grade 3 complications in the CPAP + 4 group (23.5% vs. 0%, *p* = 0.036). No significant differences were found in the effectiveness of oxygenation, ventilation, or pulmonary ablation.

**Conclusion:**

CPAP is unsafe during CT-guided RFA for lung cancer under PSA even at the lowest pressure setting.

**Trial registration:**

ClinicalTrials.Gov, ClinicalTrials.gov ID NCT02117908, Registered 11 April 2014, https://www.clinicaltrials.gov/study/NCT02117908

**Critical relevance statement:**

This study highlights the hazards of continuous positive airway pressure during radiofrequency ablation of lung cancer, even at minimal pressures, deeming it unsafe under procedural sedation and analgesia in pulmonary interventional procedures. Findings provide crucial insights to prioritise patient safety.

**Key Points:**

- No prior randomised controlled trials on CPAP safety in percutaneous lung thermo-ablation.
- Standardised outcome measures are crucial for radiology research.
- CPAP during lung RFA raises hospital stay and the risk of complications.
- CPAP is unsafe during CT-guided RFA of lung cancer under procedural sedoanalgesia.

**Graphical Abstract:**

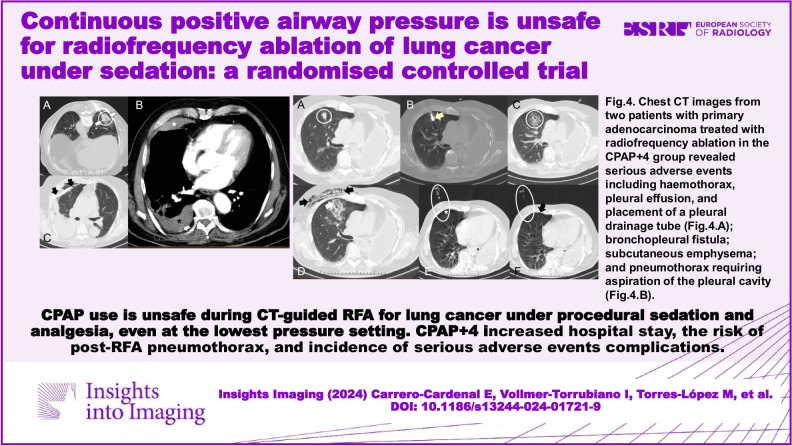

## Introduction

Lung cancer is the leading cause of cancer-related death [1]. Computed tomography (CT)-guided percutaneous radiofrequency ablation (RFA) is a minimally invasive therapeutic option for lung cancer when surgery is contraindicated [2]. It is also the most frequently reported ablation technique for the treatment of lung cancer [3]. Pulmonary RFA is a high-risk interventional procedure because patient’ comorbidities and the potential hazards of the procedure itself [4]. Major complications range from 2% to 10%, occur mainly in the first month and can be life-threatening [5]. Procedural sedation and analgesia (PSA) have become the most commonly used anaesthetic technique [6, 7].

Atelectasis is a common pulmonary complication associated with PSA levels. A previous study observed up to 63% atelectasis on thoracic CT scans of patients with lung cancer treated with RFA under PSA, with over half of the patients requiring an increase in oxygen flow to maintain adequate intraoperative oxygenation [8]. Pulmonary atelectasis during lung RFA under PSA can lead to lesion retraction towards the pulmonary hilum, increasing the risk of bronchial or vascular injury or incomplete treatment [9]. Moreover, atelectasis can impair respiratory function, increase the likelihood of postoperative pulmonary complications, and worsen the clinical outcomes [10].

Continuous positive airway pressure (CPAP) reduces pulmonary atelectasis associated with anaesthesia, helps maintain functional residual capacity, improves V/Q matching and oxygenation, and avoids airway collapse associated with obstructive apnoea [11]. However, performing pulmonary RFA with positive airway pressure may increase the frequency or severity of RFA complications.

The risk-benefit of applying CPAP in patients with lung cancer undergoing RFA under PSA is unknown. To our knowledge, no previous randomised controlled trials (RCT) have been published on the use of CPAP in percutaneous lung thermo-ablation. We designed this pilot study to assess the safety of a minimum CPAP of 4 cm H_2_O applied during CT-guided percutaneous pulmonary RFA of malignant lung tumours under PSA.

## Methods and study design

We conducted a single-centre, prospective, randomised, parallel-group, placebo-controlled study with an open-label medical device. Ethical approval (HCP/2013/159) was granted by the Clinical Research Ethics Committee of the Hospital Clinic Barcelona, Barcelona, Spain (Chairperson Dr. X. Carné Cladellas) on 16 September 2013. The study was authorised by the Spanish Agency of Medicines and Medical Devices (AEMPS) on 24 March 2014 and registered at ClinicalTrials.gov (NCT02117908) on 11 April 2014. The study was conducted in accordance with the Declaration of Helsinki, and written informed consent was obtained from all patients.

### Participants

We considered patients over 18 years of age scheduled for CT-guided RFA of a malignant pulmonary tumour under PSA who were capable of undergoing the tests and explorations required for the study as eligible. The exclusion criteria were any radiological contraindication for RFA, lung nodule biopsy just prior to RFA, intercurrent disease, inability to understand the procedure or intolerance to the CPAP test. We also excluded patients scheduled for additional pulmonary RFA.

### Randomisation, interventions and blinding

We randomised the patients to receive CPAP + 4 or a modified mask for placebo CPAP (Sham-CPAP). We used a computerised randomisation list prepared by an independent investigator for the allocation. The randomisation sequence was generated using the SAS^®^ 9.2 PROC PLAN procedure (SAS Institute, Inc, Cary, North Carolina) with a 1:1 allocation using a random block size of 4. The principal investigator, who was unaware of the randomisation sequence, included cases that met the inclusion criteria in the electronic case report form (eCRF) where the randomisation number appeared indicating CPAP + 4 or Sham-CPAP. We employed a CPAP device (ResMed S9, ResMed Ltd., Bella Vista, New South Wales, Australia) with a fixed pressure of 4 cmH_2_O and a full-face mask (Ultra MirageTM NV full-face mask, ResMed Ltd., Bella Vista, New South Wales, Australia) in the CPAP + 4 group. The Sham-CPAP group received a placebo treatment described by Farré et al [12]. We launched the CPAP or Sham device when PSA was started. The patients received oxygen at a flow rate of 3 L min^−1^. The patients and radiologists were blinded to the group allocations.

### Study outline

**Fig. 1:**
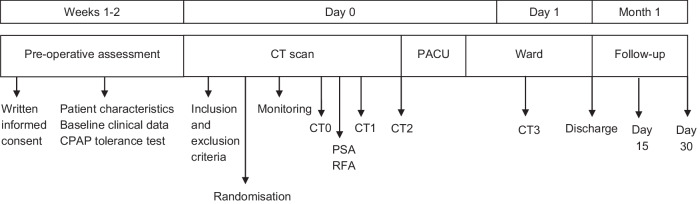
Study protocol outline. CPAP, continuous positive airway pressure; CT, computed tomography; CT0, CT scan before the start of the procedure and PSA once the patient was positioned; CT1, CT scan at the end of the procedure, electrode inserted, before stopping PSA; CT2, CT scan after removal of the ablation electrode and PSA, patient in supine decubitus, awake, CPAP mask removed; CT3: control CT scan at 24 h prior to discharge; Day 0-, day of the pulmonary RFA; PACU: post-anaesthesia care unit; PSA, procedural sedation and analgesia; RFA, radiofrequency ablation

Figure 1 presents an outline of the study. We established the CPAP + 4 cancellation criterion as any clinically relevant complication attributable to the device.

### Monitoring

**Fig. 2:**
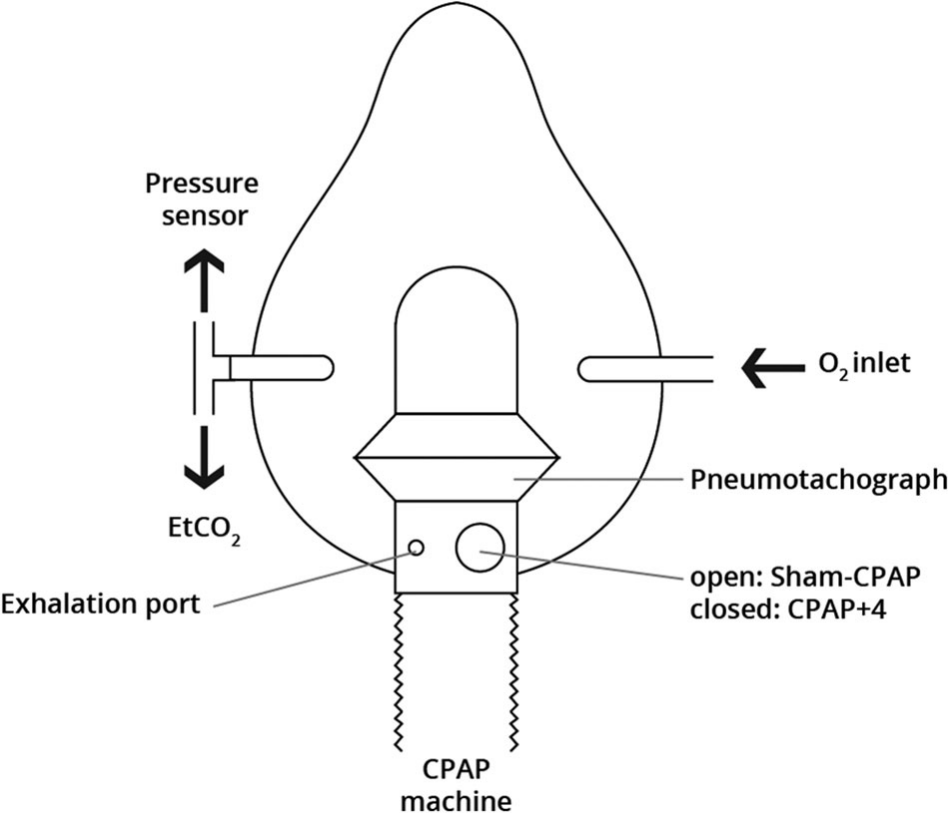
Wiring diagram of the mask for CPAP + 4 or Sham-CPAP. A pressure sensor (Honeywell S&C, Minneapolis, USA) was connected to one of the mask’s inlets, and a pneumotachograph (ResMed Ltd., Bella Vista, New South Wales, Australia) was placed between the CPAP tube and the mask. CPAP pressure and respiratory flow values were measured and recorded to verify nasal pressure and ensure the correct estimation of apnoea and hypopnoea episodes. In the other inlet of the mask, a probe connected to a capnograph (Capnostream® 20 P Oridion, Jerusalem, Israel) was placed to record EtCO_2_ and respiratory rate. CPAP + 4, continuous positive airway pressure of 4 cm H_2_O; EtCO_2_, end-tidal CO_2_; O_2_, oxygen; Sham-CPAP, modified mask for placebo CPAP

Respiratory monitoring included airway pressure, respiratory airflow, end-tidal carbon dioxide (ETCO_2_), respiratory rate (RR) (Fig. 2), and peripheral oxygen saturation (SpO_2_). All sensors were connected to an analogue-digital converter (DATAQ^®^ Instruments) and recorded and analysed using WindAQ Data Acquisition (DAQ) software. The cut-off points were as follows: T0, basal (patient positioned); TR (1-6), end of each ablation cycle (roll-offs); T1, end of RFA, radiofrequency electrode inserted, PSA in progress, patient in RFA position and T2, RFA completed, radiofrequency electrode extracted, PSA shutdown, CPAP or Sham device removed and patient in the supine position. The mean values of the pressure (cm H_2_O), flow (L s^-1^), ETCO_2_ (kPa) and SpO_2_ (%) were calculated at 10 s around the cut-off point. The complete record (T0-T2) was analysed for episodes of apnoea (absence of respiratory flow for 10 s or more), hypopnoea (30% reduction in respiratory flow for 10 s or more), hypoxaemia (SpO_2_ < 90%) or hypercapnia (ETCO_2_ > 5.3 kPa). The percentage of recording time in which SpO_2_ was below 90% (CT90), and ETCO_2_ was higher than 5.3 kPa (40 mmHg) was calculated.

Patient monitoring also included electrocardiography using a 3-lead system (Philips IntelliVue MP50, Soma Technology, Inc. Bloomfield, USA), continuous non-invasive haemodynamic monitoring (Nexfin^®^, BMEYE, Amsterdam, The Netherlands), tympanic temperature (tympanic temperature sensor TTS-400, Smiths Medical, Minnesota, USA), Bispectral Index (BIS), (BIS^TM^, Medtronic, formerly Covidien, Minneapolis, USA), Ramsay sedation scale [13] and the visual analogue scale (VAS), (0–10). The monitoring parameters were recorded at each cut-off point.

### CT-guided percutaneous pulmonary RFA

A Somatom^®^ Emotion Duo CT scanner (Siemens^®^, Erlangen, Germany) was used in this study. The CT protocol for RFA performed at our hospital remained unchanged. Each patient underwent four CT acquisitions (Fig. 1), with 5 mm thick reconstructions using H80 and H30 filters, as well as 1.5 mm thick reconstructions using an H30 filter. All reconstructions were saved in picture archiving and communication systems (PACS) for further analysis. Data analysis was performed using the Pulmo 3D SyngoVia^®^ software (Siemens^®^, Erlangen, Germany).

We recorded the duration of the pulmonary RFA procedure, maximum radiofrequency generator power (W), generator impedance (Ohm) and thermal ablation generator time (s). Patients were positioned in the supine, prone, or lateral decubitus position with their arms extended depending on the most appropriate imaging approach. Following asepsis and notching, local anaesthetic infiltration was performed at the puncture site. The radiofrequency generator, which uses a feedback system based on electrical impedance, automatically determines the end of the ablation cycles (roll-offs). Two types of ablation generators and electrodes were used: the RF3000^TM^ radiofrequency generator and the LeVeen CoAccess^TM^ electrode system with a coaxial needle placement system, a self-expanding electrode, and the option to use three sizes (3, 3.5, and 4 cm), (Boston Scientific, Natick, Massachusetts, USA) or the Cool-tip^TM^ RF ablation generator and the Cool-tip^TM^ RF ablation system with a single needle system, cooled tip and the possibility of using a 2 or 3 cm effective tip (Medtronic, formerly Covidien, Minneapolis, USA).

### Anaesthesia

For PSA, we administered a target-controlled infusion (TCI) (Fresenius Kabi Orchestra^®^ Base Primea Bad Homburg, Germany) of remifentanil supplemented with TCI of propofol and an intravenous bolus of ketamine (5–10 mg) as determined by the anaesthesiologist. PSA was initiated after TC0 in conjunction with Sham-CPAP or CPAP + 4 and stopped after TC1. If the patient experienced episodes of hypoxaemia (SpO_2_ < 90%), the supplemental oxygen flow was increased. Increases in ETCO_2_ > 5.3 kPa were tolerated as long as there were no clinical repercussions. Adverse respiratory events were managed based on the clinical judgement of the attending physicians. Incremental doses of 5 mg IV urapidil were administered if the mean arterial pressure (MAP) increased above 20% and incremental doses of ephedrine (5 mg) or phenylephrine (50 μg) were administered if MAP fell below 20% of baseline. The infusion time delivered by the TCI pump and anaesthetic drug doses were recorded. After completion of the procedure, patients were monitored in the post-anaesthesia care unit (PACU) and transferred to the hospital ward. Discharge was expected within 24 h of the CT3 scan.

### Outcomes

The primary outcomes were the number (%) of subjects reporting at least one serious adverse event (SAE), the Classification for complications from the Cardiovascular and Interventional Radiological Society of Europe (CIRSE) classification system for complications of interventional radiology [14] the Clavien-Dindo classification of surgical complications [15], hospital stays and readmissions. Secondary outcomes included adverse events (AEs), intraoperative episodes of hypopnoea or apnoea, minimum SpO_2_, CT90, maximum ETCO_2_, percentage of time ETCO_2_ > 5.3 kPa, airway interventions, and the local radiological efficacy of RFA (a complete tumour ablation margin, a minimum halo thickness of 5 mm and an increase in tumour size from CT0 to CT3). SAEs and AEs were reported and coded according to the MedDRA (https://www.meddra.org/how-to*use/basics/hierarchy*) and MDCG 2020-10/1 guidelines (https://ec.europa.eu/health/system/files/2020-09/md_mdcg_2020-10-1_guidance_safety_reporting_en_0.pdf). SAE and AE reporting were conducted from recruitment to one-month follow-up.

### Sample size

No previous studies have applied the proposed anaesthetic technique with the same type of target population. Similar studies [16, 17] have used sample sizes of approximately 20 patients per group. A sample size of 22 patients per group was calculated considering a 10% loss or no consent.

### Statistical analysis

The statistical analysis plan was approved by the authors before the analysis began. Intention-to-treat (ITT) and per-protocol (PP) analyses were performed. Continuous variables were reported as mean ± SD or median (IQR). Categorical variables are presented as number of cases (*n*) and percentages (%). Comparison of continuous variables was performed using the Student’s *t*-test or Mann–Whitney test, as appropriate for parametric and non-parametric variables. Categorical variables were compared using Fisher’s exact test. For binary variables, the odds ratio (OR) and their 95% confidence interval (95%CI) were estimated to assess the risk of complications using a logistic regression model. If the risk could not be estimated, differences between treatments were compared using Fisher’s exact test.

Longitudinal continuous variables were analysed using mixed models for repeated measures (MMRM). Statistical analysis was performed using SAS version 9.4 or higher (SAS Institute Inc., Cary, NC, USA) and statistical significance was established at the two-sided 5% level.

## Results

**Fig. 3:**
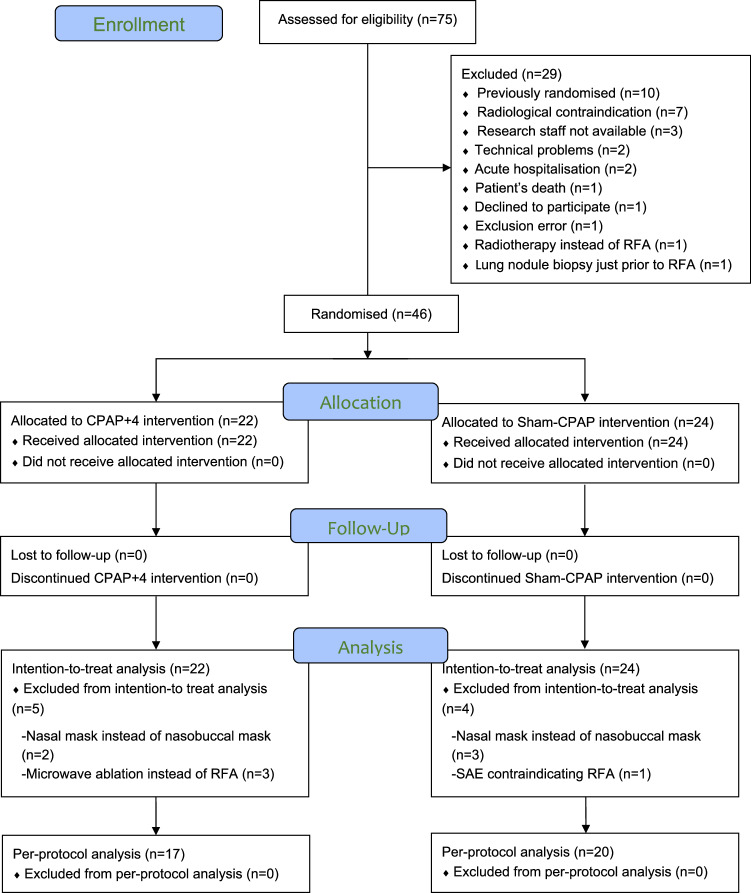
CONSORT flow diagram. CPAP + 4, continuous positive airway pressure of 4 cm H_2_O; RFA, radiofrequency ablation; SAE, serious adverse event; Sham-CPAP, modified mask for placebo CPAP. Follow-up visits were conducted at discharge and on days 15 and 30 after RFA. The intention-to-treat (ITT) population included all subjects who were randomised, and the per-protocol (PP) population included cases that adhered to the protocol

Figure 3 illustrates the flow of the participants. Patient enrolment was conducted between 7 November 2014 and 21 February 2018, in the Radiological Department. Table 1 summarises patient characteristics and baseline clinical data.

**Table 1:** Patient characteristics and baseline clinical data

	ITT population			PP population		
	CPAP + 4 *n* = 22	Sham-CPAP *n* = 24	Total *n* = 46	CPAP + 4 *n* = 17	Sham-CPAP *n* = 20	Total *n* = 37
Characteristic						
Age, years	71.8 ± 11.4	75.8 ± 9.7	73.9 ± 10.6	73.4 ± 9.8	76.2 ± 8.6	74.9 ± 9.1
Male	13 (59.1)	18 (75)	31 (67.4)	11 (64.7)	14 (70)	25 (67.6)
Female	9 (40.9)	6 (25)	15 (32.6)	6 (35.3)	6 (30)	12 (32.4)
Weight, kg	74.6 ± 13.8	72.8 ± 12.4	73.7 ± 13.01	75.4 ± 13.8	71.1 ± 12.3	73.2 ± 13.07
Height, cm	164.8 ± 12.01	165.1 ± 7.8	165 ± 9.9	165.1 ± 11.4	163.8 ± 7.1	164.4 ± 9.2
BMI, kg m^−2^	27.5 ± 4.5	26.7 ± 4.5	27.1 ± 4.4	28.1 ± 4.4	26.8 ± 4.6	27.4 ± 4.5
ASA physical status						
2	0 (0)	1 (4.2)	1 (2.2)	0 (0)	1 (5)	1 (2.7)
3	18 (81.8)	16 (66.7)	34 (73.9)	14 (82.4)	14 (70)	28 (75.7)
4	4 (18.2)	7 (29.2)	11 (23.9)	3 (17.6)	5 (25)	8 (21.6)
Active smoker	4 (18.2)	5 (20.8)	9 (19.6)	3 (17.6)	3 (15)	6 (16.2)
Ex-smoker	15 (68.2)	16 (66.7)	31 (67.4)	12 (70.6)	14 (70)	26 (70.3)
Medical history						
COPD	7 (31.8)	14 (58.3)	21 (45.6)	4 (23.5)	11 (55)	15 (40.5)
Respiratory symptoms	18 (81.8)	20 (83.3)	38 (82.6)	14 (82.4)	16 (80)	30 (81.8)
Previous lung surgery	11 (50)	7 (29.2)	18 (39.1)	8 (47.1)	5 (25)	13 (35.1)
Sleep apnoea	3 (13.6)	2 (8.3)	5 (10.9)	2 (11.8)	2 (10)	4 (10.8)
Nocturnal CPAP	2 (9.1)	1 (4.2)	3 (6.5)	2 (11.8)	1 (5)	3 (8.1)
Heart failure	1 (4.5)	2 (8.3)	3 (6.5)	1 (5.9)	1 (5)	2 (5.4)
Arterial hypertension	15 (68.2)	15 (62.5)	30 (65.2)	13 (76.5)	14 (70)	27 (72.9)
Coronary heart disease	3 (13.6)	3 (12.5)	6 (13.0)	3 (17.6)	2 (10)	5 (13.5)
Diabetes mellitus	4 (18.2)	6 (25)	10 (21.7)	3 (17.6)	5 (25)	8 (21.6)
Basal tests at recruitment						
SpO_2_%	97.68 ± 1.09	97.42 ± 1.21	97.54 ± 1.15	97.91 ± 1.05	97.40 ± 1.31	97.54 ± 1.19
Positive cough test	1 (4.5)	10 (41.7)	11 (23.9)	1 (5.9)	8 (40)	9 (24.3)
Obstructive spirometry pattern	11 (50)	16 (66.7)	27 (58.7)	9 (52.9)	12 (60)	21 (56.7)
Lung cancer to be treated						
Primary lung cancer	6 (27.3)	13 (54.2)	19 (41.3)	5 (29.41)	10 (50)	15 (40.54)
Lung metastases	16 (72.7)	11 (45.8)	27 (58.7)	12 (70.59)	10 (50)	22 (59.46)
Lung cancer location						
Left lung superior lobe	6 (27.3)	2 (8.3)	10 (21.7)	7 (41.2)	2 (10)	9 (24.3)
Left lung inferior lobe	4 (18.2)	3 (12.5)	7 (15.2)	2 (11.8)	2 (10)	4 (10.8)
Right lung superior lobe	3 (13.6)	10 (41.7)	13 (28.3)	3 (17.7)	8 (40)	11 (29.8)
Right lung middle lobe	1 (4.5)	0 (0)	1 (2.2)	1 (5.9)	0 (0)	1 (2.7)
Right lung inferior lobe	7 (31.8)	9 (37.5)	16 (34.8)	5 (26.3)	8 (40)	13 (35.1)
Proximity to pleura	11 (50)	11 (45.8)	22 (47.8)	7 (41.2)	9 (45)	16 (43.2)

Categorical variables are expressed as the number of cases (%) and continuous variables as mean ± SD. The demographic variables had no missing data

*ASA* American Society of Anaesthesiologists physical status, *BMI* body mass index, *COPD* chronic obstructive pulmonary disease, *CPAP* *+* *4* continuous positive airway pressure of 4 cm H_2_O, *ITT* intention-to-treat, *PP* per-protocol, *RFA* radiofrequency ablation, *Sham-CPAP* modified mask for placebo CPAP, *SpO*_*2*_ oxygen saturation

Airway pressure monitoring confirmed the difference in the mean airway pressure values set for each study group (see Table, Supplemental Digital Content 1, which displays MMRM analysis of airway pressure and respiratory flow). The groups were comparable, except for the maximum power supplied by the RF generator and MMRM-estimated BIS (see Table, Supplemental Digital Content 2, for results relating to patient positioning, duration of procedure, anaesthetic and RFA parameters, and monitoring).

The CPAP + 4 group had a longer hospital stay (Table 2). The risk of AE in the ward post-RFA was 4-fold higher in the CPAP + 4 group (OR (95%CI), ITT, 4.250 (1.234 to 14.637), *p* = 0.021; PP, 4.457 (1.110 to 17.899), *p* = 0.035), with a higher incidence of pneumothorax (ITT, *n* = 5/22 (22.7%) vs. *n* = 0/24 (0%), *p* = 0.019; PP, *n* = 5/17 (29.4%) vs. *n* = 0/20 (0%), *p* = 0.014).

**Table 2:** Primary outcomes of the study

	ITT population					PP population				
	CPAP + 4 *n* = 22	Sham-CPAP *n* = 24	OR (95%CI)	*p-* value	Fisher’s exact test	CPAP + 4 *n* = 17	Sham-CPAP *n* = 20	OR (95%CI)	*p-* value	Fisher’s exact test
Serious adverse event	5 (22.7)	1 (4.2)	6.76 (0.72 to 63.33)	0.093	0.089	4 (23.5)	0 (0)	NA	NA	0.036
Radiological classification of complications (CIRSE)										
Grade 1	22 (100)	23 (95.8)	NA	NA	1.000	17 (100)	20 (100)	NA	NA	NA
Grade 3	4 (18.2)	1 (4.2)^a^	5.33 (0.53 to 52.73)	0.152	0.178	4 (23.5)	0 (0)	NA	NA	0.036
Grade 4	1 (4.5)^b^	0 (0)	NA	NA	0.478	0 (0)	0 (0)	NA	NA	NA
Surgical classification of complications (Clavien-Dindo)										
Grade I	22 (100)	23 (95.8)	NA	NA	1.000	17 (100)	20 (100)	NA	NA	NA
Grade II	19 (86.4)	17 (70.8)	2.13 (0.45 to 10.09)	0.339	0.289	14 (82.4)	15 (75.0)	1.55 (0.31 to 7.75)	0.589	0.701
Grade III-a	4 (18.2)	1 (4.2)	5.33 (0.53 to 52.73)	0.152	0.177	3 (17.6)	1 (5)	4.07 (0.38 to 43.36)	0.544	0.315
Grade IV-a	0 (0)	1 (4.2)	NA	NA	1.000	0 (0)	0 (0)	NA	NA	NA
Inpatient nights	1.5 ± 1.1	1 ± 0			0.022	1.6 ± 1.2	1 ± 0			0.036
Prolonged hospital stay	5 (22.7)	0 (0)	NA	NA	0.021	4 (23.5)	0 (0)	NA	NA	0.036
Readmissions	0 (0)	0 (0)	NA	NA	NA	0 (0)	0 (0)	NA	NA	NA

Categorical variables are expressed as the number of cases (% of total cases) and continuous variables as mean ± SD

*CI* confidence interval, *CIRSE* Cardiovascular and Interventional Radiological Society of Europe, *CPAP* *+* *4* continuous positive airway pressure of 4 cm H_2_O, *ITT* intention-to-treat, *NA* not applicable, *OR* odds ratio, *PP* per-protocol, *RFA* radiofrequency ablation, *Sham-CPAP* modified mask for placebo CPAP, *CIRSE grade 1* complications during the procedure that could be resolved within the same session; no additional therapy, no post-procedure sequelae, no deviation from the normal post-therapeutic course, *CIRSE grade 3* additional post-procedure therapy or prolonged hospital stay, no post-procedure sequelae, *CIRSE grade 4* complications causing permanent mild sequelae (resuming work and independent living), *Clavien-Dindo grade I* any deviation from the normal postoperative course without the need for pharmacological treatment or surgical, endoscopic or radiological interventions. Acceptable therapeutic regimens include antiemetics, antipyretics, analgesics, diuretics, electrolytes, and physiotherapy. This grade also includes wound infections opened at the bedside, *Clavien-Dindo grade II* complications requiring pharmacological treatment with drugs other than those allowed for grade I complications. Blood transfusions and total parenteral nutrition were also included, *Clavien-Dindo grade III-a* complications requiring surgical, endoscopic, or radiological intervention, intervention not under general anaesthesia, *Clavien-Dindo grade IV-a* life-threatening complications (including central nervous system complications) requiring intermediate care/intensive care unit management, and single organ dysfunction (including dialysis)

^a^ Sham-CPAP case CIRSE grade 3 was recorded after the study ended

^b^ CPAP + 4 case CIRSE grade 4 also involved a prolonged hospital stay (the CIRSE classification allows only one option to be selected)

**Fig. 4:**
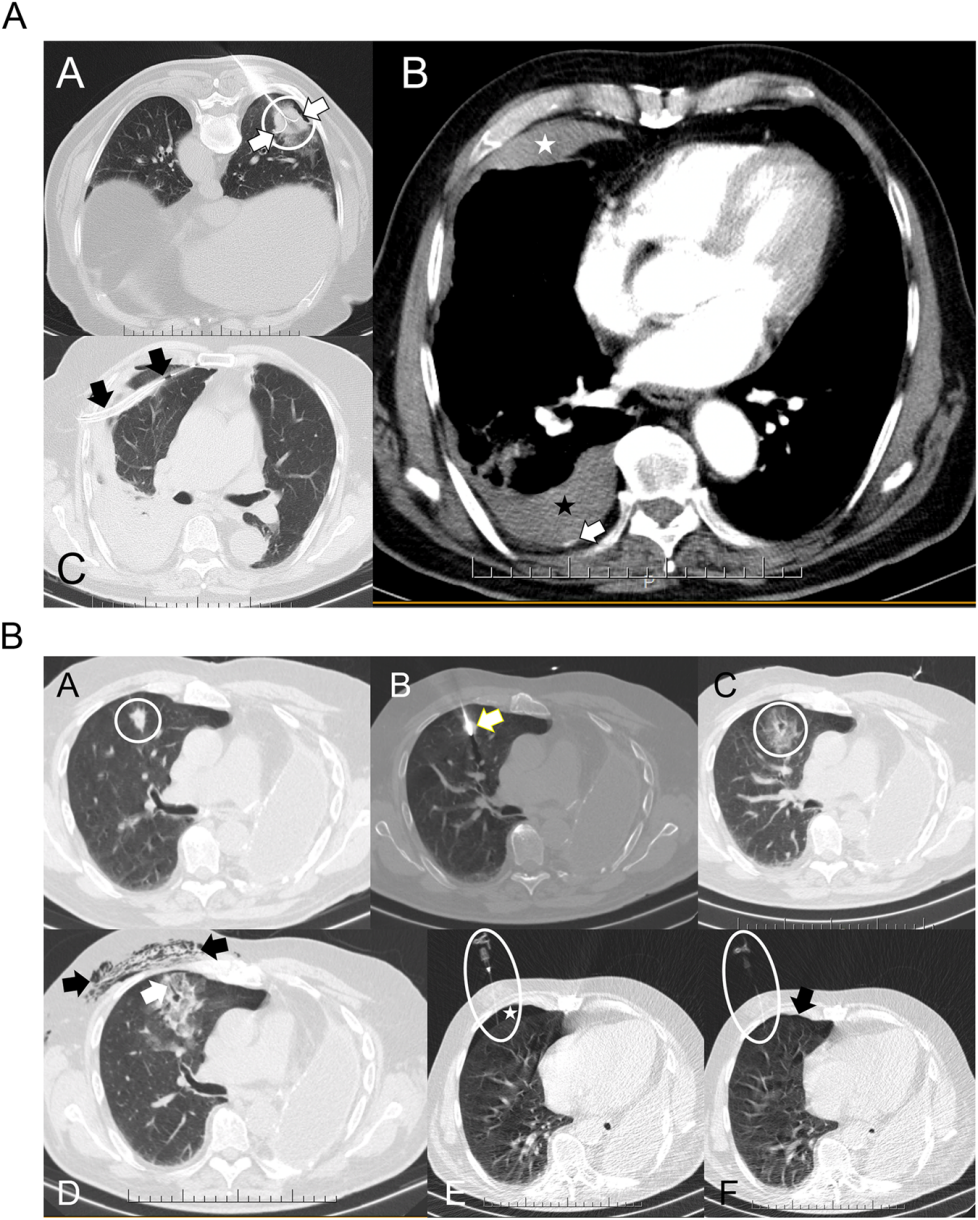
**A**, **B** Chest CT images from two patients with primary lung adenocarcinoma treated with RFA in the CPAP + 4 group who developed SAEs. **A** Thoracic CT images in the axial plane in lung (A, C) and mediastinal (B) windows. A: Radiofrequency needle with expanded electrodes (white arrows) inside the lung nodule (white circle). B: Post-procedure haemothorax. Due to the prone position during treatment, increased density can be seen in the anterior portion of the pleural cavity corresponding to haemothorax (white star) and pleural effusion in the posterior part (black star). A small bleeding point can also be seen in the parietal pleura (black arrow). C: Scan was performed to verify the correct placement of a pleural drainage tube (black arrows). **B** Thoracic CT images in the axial plane and lung window. A: Lung nodule in the anterior segment of the right upper lobe (white circle) in a patient with left pneumonectomy. B: Radiofrequency needle inside the lung nodule (white arrow). C: Adequate post-procedure tumour ablation margin (white circle). D: Linear air leak (white arrow) extending from the nodule to the pleura, corresponding to a post-treatment bronchopleural fistula. Subcutaneous emphysema is also evident (black arrows). E: Given the presence of a mild pneumothorax (white star) and the history of previous pneumonectomy, it was decided to aspirate the pneumothorax (white ellipse). F: After aspiration of the pleural cavity and with the needle still inside (white ellipse), the pneumothorax is almost completely resolved (black arrow). CT, computed tomography; CPAP + 4, continuous positive airway pressure of 4 cm H_2_O; RFA, radiofrequency ablation. The patients provided written consent for the use of medical images

**Fig. 5:**
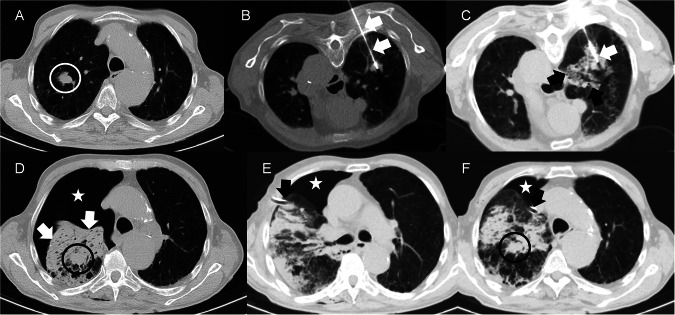
Chest CT images showing SAEs in a patient from the Sham-CPAP group. Non-small-cell lung carcinoma in a patient from the Sham-CPAP group in whom radiofrequency ablation was contraindicated due to pulmonary haemorrhage following insertion of the radiofrequency needle. Chest CT images are shown in the axial plane and lung window. **A** Lung nodule in the apical segment of the right upper lobe (white circle). **B** Radiofrequency needle (white arrows) inside the pulmonary nodule with prone posterior access. **C** Moderate haemorrhage around the radiofrequency needle (white arrow) extending to the rest of the lobe (black arrows). **D** Significant pulmonary haemorrhage (white arrows) surrounding the nodule (black circle) and marked pneumothorax (white star). **E** Verification of the placement of the pleural drainage tube with anterolateral access (black arrow) and decrease in the volume of the pneumothorax (white star). **F** End of the drainage tube in a paramediastinal position (black arrow), a decrease in the volume of the pneumothorax (white star), re-aired right lung parenchyma, and nodule more clearly visualised (black circle). CT, computed tomography; Sham-CPAP, modified mask for placebo CPAP; RFA, radiofrequency ablation. The patient provided written consent for the use of medical images

PP analysis also showed a higher incidence of SAEs and CIRSE grade 3 complications in the CPAP + 4 group (Table 2). SAEs in the CPAP + 4 group included chest drainage (three patients), pneumothorax, hypoxaemia, dyspnoea, postoperative respiratory failure (two patients), pleural cavity aspiration, pleural effusion, subcutaneous emphysema, pleural fistula, and haemothorax (one patient), (Fig. 4A, B). The SAEs in the Sham-CPAP group were pulmonary haemorrhage, hypoxaemia, pneumothorax, and chest drainage (one patient, Fig. 5).

All patients presented with at least one AE. The most frequent AEs were hypercapnia, chest pain (pain at the RFA site, VAS > 0), pain in extremities (arm positional pain, VAS > 0), clinical or radiological pneumothorax, hypopnoea, and hypoxaemia. Sweating was 10 times more at risk in the Sham-CPAP group than in the CPAP + 4 group (see Table, Supplemental Digital Content 3, which lists the incidence of adverse events registered). Severe positional arm pain (VAS > 7) was reported 2.3 times more frequently than severe pain at the thoracic RFA site, with no difference between the groups (see Table, Supplemental Digital Content 4 for the maximum VAS values).

No differences were found between groups in the number or duration of apnoea or hypopnoea episodes, the minimum SpO_2_, the maximum ETCO_2_, the CT90, the percentage of time ETCO_2_ above 5.3 kPa and the number of patients who needed verbal or physical stimulation to reverse respiratory depression (Table 3). None of the patients required chin tilt, jaw thrust, nasopharyngeal or oropharyngeal cannula insertion, laryngeal mask, or tracheal intubation.

**Table 3:** Secondary outcomes. Respiratory parameters

	ITT population				PP population			
Variable	CPAP + 4 *n* = 22	Sham-CPAP *n* = 24	Total *n* = 46	*p*-value	CPAP + 4 *n* = 17	Sham-CPAP *n* = 20	Total *n* = 37	*p*-value
Number of apnoea episodes	7.6 ± 6.7	6.1 ± 4.1	7 ± 5.6	0.609	8.1 ± 7.06	6.1 ± 4.1	7.2 ± 5.7	0.528
Duration of apnoea episodes, min	2.1 ± 1.6	2.6 ± 2.09	2.3 ± 1.7	0.613	2.1 ± 1.7	2.6 ± 2.09	2.3 ± 1.8	0.671
Number of hypopnea episodes	3.3 ± 3.2	5.4 ± 5.5	4.5 ± 4.7	0.276	3.5 ± 3.4	5.4 ± 5.5	4.6 ± 4.8	0.331
Duration of hypopnea episodes, min	2 ± 2.6	4.2 ± 7.5	3.3 ± 6.0	0.352	2.1 ± 2.7	4.2 ± 7.5	3.4 ± 6.1	0.399
Minimum SpO_2_, %	85.5 ± 9.1	87.7 ± 6.4	86.7 ± 7.8	0.365	84.8 ± 9.4	88.6 ± 6.1	86.9 ± 7.9	0.152
Maximum EtCO_2_, kPa	6.1 ± 0.7	6.2 ± 0.7	6.2 ± 0.7	0.953	6.1 ± 0.7	6.2 ± 0.7	6.2 ± 0.7	0.709
CT90, %	2.2 ± 4.6	0.6 ± 0.9	1.3 ± 3.3	0.108	2.09 ± 4.5	0.5 ± 1.0	1.2 ± 3.2	0.185
Percentage of time EtCO_2_ > 5.3 kPa, %	3.7 ± 7.9	4.8 ± 7.02	4.2 ± 7.4	0.627	2.2 ± 3.8	5.1 ± 7.1	3.7 ± 5.8	0.139
Actions against respiratory depression								
Reduced TCI dose	4 (18.1)	4 (16.6)	8 (17.3)	1.000	4 (23.5)	3 (15)	7 (18.9)	0.680
Increased FiO_2_	6 (27.2)	6 (25)	12 (26.09)	1.000	5 (29.4)	4 (20)	9 (24.3)	0.703
Verbal stimulation required	8 (36.3)	5 (20.8)	13 (28.2)	0.330	6 (35.2)	3 (15)	9 (24.3)	0.250
Physical stimulation needed	1 (4.5)	1 (4.1)	2 (4.3)	1.000	0 (0)	1 (5)	1 (2.7)	1.000

Categorical variables are expressed as the number of cases (% of total cases) and continuous variables as mean ± SD

*CPAP* *+* *4* continuous positive airway pressure of 4 cm H_2_O, *CT90* percentage of recording time in which SpO_2_ was below 90%, *ETCO*_*2*_ end-tidal CO_2_, *FiO*_*2*_ fraction of inspired oxygen, *ITT* intention-to-treat, *Percentage of time EtCO*_*2*_ *>* *5.3* *kPa* percentage of recording time in which EtCO_2_ was higher than 5.3 kPa (40 mmHg), *PP* per-protocol, *Sham-CPAP* modified mask for placebo CPAP, *SpO*_*2*_ oxygen saturation measured by pulse oximeter, *TCI* target-controlled infusion

The local radiological efficacy of RFA was similar in both groups (see Table, Supplemental Digital Content 5, which presents CT measurements of the tumour ablation margin, minimum halo thickness, and change in tumour size).

## Discussion

The application of minimal CPAP to patients with malignant lung neoplasms treated with RFA under PSA and spontaneous breathing is unsafe. A 4 cm H_2_O airway pressure prolongs hospital stay, increases the risk of AE in the ward, mainly pneumothorax, increases the incidence of SAE and CIRSE grade 3 complications, and does not improve respiratory function.

To our knowledge, this is the first RCT to analyse the effects of CPAP during pulmonary RFA. Only isolated cases have been previously reported. Nachiappan et al [18]. published a case of bronchopleural fistula following the use of bi-level positive airway pressure (BiPAP). In our series, one patient in the CPAP + 4 group developed a bronchopleural fistula post-treatment. We agree that the use of BiPAP or CPAP devices in these patients is an additional risk factor for the occurrence of this complication. Elliott et al [19]. support the application of CPAP in intervening isolated lung during RFA under general anaesthesia with one-lung ventilation because CPAP avoids complete lung collapse, decreases the risk of the radiofrequency catheter approaching the pulmonary hilum, and consequently, the risk of bronchial or vascular injury. We minimised this potential hazard because we did not apply general anaesthesia and mechanical ventilation, two known factors favouring lung collapse [20], and the level of sedation achieved during PSA was neither deep nor prolonged enough to promote atelectasis [21]. We attribute our lower incidence of atelectasis to the use of the TCI system for remifentanil, compared with other techniques of conscious sedation for RFA of lung cancer [8].

The incidence of pneumothorax in spontaneously breathing patients treated with CPAP is unknown, although respiratory pathology underlies most published cases [22]. The main mechanism is alveolar overdistension caused by the continuous pressure in the airway [23]. On the other hand, pneumothorax is already the most frequent complication of pulmonary RFA as a consequence of pleural perforation of the RF needle [3, 24, 25]. CPAP increased the occurrence of pneumothorax with clinical repercussions that required chest drainage and prolonged hospitalisation, as well as pneumothoraces detected after the procedure. Although a minimum airway pressure of 4 cm H_2_O would not justify alveolar overdistension as the main mechanism of pneumothorax, we consider the application of CPAP in pulmonary RFA procedures, where patients have underlying respiratory pathology, as an additional risk factor.

Three recent studies [16, 17, 26] show that applying CPAP 5–10 cm H_2_O at an oxygen flow of 5–10 L min^-1^ in procedures under deep sedation decreased the need for airway interventions, incidence and severity of hypoxaemia and hypercapnia and frequency and duration of apnoea and hypopnoea episodes. We did not find these beneficial effects for several reasons: (1) we limited the CPAP pressure to a minimum of 4 cm H_2_O, (2) the oxygen flow rate we applied was much lower, (3) the body mass index of our patients was lower,(4) only five of our patients had obstructive sleep apnoea and (5) our PSA technique was based on the use of remifentanil instead of propofol. Remifentanil exerts its depressant effect on the neurons of the respiratory centre, which is partly counteracted by the stimulatory effect of the resulting CO_2_ and hypoxaemia on chemoreceptors [27] In contrast, propofol increases airway collapsibility by reducing genioglossus muscle activity in proportion to the concentration of propofol and depth of sedation [28]. This may explain why, despite the high incidence of apnoea, hypopnoea, hypercapnia, and hypoxaemia recorded, these episodes were of short duration.

The application of CPAP + 4 had a protective effect on profuse sweating. We do not know the reason for this finding because thermal stimuli, remifentanil dosage, sedation and analgesia level, and body temperature were similar in both groups. Although ETCO_2_ values were not different between the groups, small, non-significant differences in ETCO_2_ values could explain this.

Our findings support the applicability of the CIRSE classification for discerning between AEs and SAEs in safety trials and underscore the limitations of the Clavien-Dindo classification in categorising interventional radiology complications. Registration of AEs according to European regulations was not effective in discriminating severity. Our findings reaffirm the importance of recording complications based on the outcomes and severity of the sequelae. In this sense, hospital stay proved to be a valuable primary outcome. We did not find any published data on the incidence of pulmonary complications classified as SAE to compare our results.

Our study had several limitations. First, it was conducted at a single-centre, and we cannot generalise our results. Second, although the sample size was sufficient to demonstrate the risk of applying CPAP, this was a pilot study and our results must be interpreted with caution. We could not perform a post-hoc analysis in specific patient populations because of insufficient caseloads. Third, we did not have an oximeter for FiO_2_ or blood gas analyses for PaO_2_ and PaCO_2_.

We do not know whether the use of high-flow nasal oxygen (HFNO), a technique of non-invasive respiratory support that delivers warmed, humidified oxygen with FiO_2_ up to 1.0 and a maximum flow rate of 60 L min^-1^, instead of CPAP in lung RFA under a PSA would be a safer procedure. We have not found any studies in which HFNO has been applied to lung RFA. We chose CPAP with a face mask instead of HFNO for two reasons: to avoid pressure loss in the airway if the patient opened his mouth, and to decrease the variability of the pressure generated, two problems associated with HFNO [29–31]. CPAP appears to be more effective in ensuring delivered pressure and reducing atelectasis, whereas HFNO improves oxygenation and reduces dead space and CO_2_ washout [32–36].

### Implications of our findings

We recommend against using CPAP for percutaneous image-guided lung ablation due to the increased risk of complications associated with its use. PSA based on remifentanil TCI, with patients maintaining spontaneous breathing, offers a safe alternative to CPAP. This technique avoids the risks associated with CPAP and does not appear to increase the incidence of atelectasis, thereby reducing the potential for vascular or bronchial injury or incomplete treatment due to lesion retraction towards the pulmonary hilum.

Furthermore, our study reinforces the importance of using standardised and validated outcome measures in interventional radiology. The effectiveness of the CIRSE classification and hospital length of stay as primary outcomes in our study supports this notion. By employing these standardised measures, we can ensure more accurate comparisons and improve the overall quality of research in this field.

## Conclusion

A minimum CPAP of 4 cm H_2_O is not safe during RFA of lung cancer under PSA and shows no beneficial effect on patient ventilation and oxygenation or the local radiological efficacy of RFA. The results of this RCT do not support the use of CPAP during RFA for lung cancer patients under PSA. Even the lowest CPAP pressure setting can be hazardous in pulmonary interventional radiology.

Multicentre, high-sample size studies are warranted to investigate the safety and efficacy of HFNO application in lung cancer patients undergoing thermal ablative procedures under PSA with remifentanil TCI. These studies should compare outcomes in patients with risk factors for respiratory complications, such as obstructive sleep apnoea, chronic obstructive pulmonary disease, and obesity, to those without such risk factors.

### Supplementary information

ELECTRONIC SUPPLEMENTARY MATERIAL

## Data Availability

The authors did not obtain explicit permission from the patients to share the research data. In order to respect the privacy rights of the patients and in accordance with the Organic Law on the Protection of Personal Data and Guarantee of Digital Rights, unfortunately, we are not allowed to share research data or materials of the study.

## Abbreviations

AE: Adverse event
BIS: Bispectral Index
CIRSE: Classification for complications from the Cardiovascular and Interventional Radiological Society of Europe
CPAP + 4: Continuous positive airway pressure of 4 cm of H_2_O
CT: Computed tomography
CT90: Percentage of time in which SpO_2_ was below 90%
ITT: Intention-to-treat
PP: Per-protocol
PSA: Procedural sedation and analgesia
RCT: Randomised controlled trial
RFA: Radiofrequency ablation
SAE: Serious adverse event
Sham-CPAP: Modified mask for placebo CPAP
TCI: Target-controlled infusion

## **Declarations**

### Author statement justifying the inclusion of nine authors

This study aimed to investigate the safety of medical devices for new indications. The study was a complex research project spanning 4 years and involved real-time data acquisition and analysis of monitoring variables. The project required the expertise of multiple individuals from different disciplines and institutions, including clinicians, researchers, and statisticians from interventional radiology, anaesthesiology, nursing, the Institute for Biomedical Research August Pi i Sunyer (IDIBAPS), the Centre for Biomedical Research in the Respiratory Diseases Network (CIBERES), and the Clinical Quality and Safety Directorate of our hospital.
