# Supplementary material for: Continuous positive airway pressure is unsafe for radiofrequency ablation of lung cancer under sedation: a randomised controlled trial
Source: Insights Imaging. 2024 Jun 20;15:153. doi: 10.1186/s13244-024-01721-9 (PMC11190131; doi:10.1186/s13244-024-01721-9)
Supplement: Supplementary file 1 — ELECTRONIC SUPPLEMENTARY MATERIAL [file 13244_2024_1721_MOESM1_ESM.pdf]

Continuous Positive Airway Pressure is Unsafe for Radiofrequency Ablation of Lung Cancer Under Sedation: A Randomised Controlled Trial.

ELECTRONIC SUPPLEMENTARY MATERIAL

Supplemental Table 1.A: Airway pressure and respiratory flow recordings. Mixed Models for Repeated Measures (MMRM). Intention-to-treat population.

Variable	Visit	Subjects		Observed values		Treatment differences Adjusted mean ± SD (95%CI)	*P*-value
		CPAP+4 N=22	Sham-CPAP N=24	CPAP+4 Mean ± SD (95%CI)	Sham-CPAP Mean ± SD (95%CI)		
Airway pressure (cm H_2O)	T0[a]	17	20	0.26 ± 0.28 (0.12 to 0.40)	0.30 ± 0.33 (0.14 to 0.46)		
Baseline = 0.007	TR1	22	21	3.34 ± 0.07 (3.21 to 3.48)	0.34 ± 0.06 (0.22 to 0.46)	3.00 ± 0.09 (2.82 to 3.18)	<.001
Treatment = <.001	TR2	20	21	3.33 ± 0.07 (3.20 to 3.47)	0.33 ± 0.12 (0.10 to 0.56)	3.00 ± 0.09 (2.81 to 3.18)	<.001
Time = <.001	TR3	6	7	3.2 7± 0.09 (3.08 to 3.45)	0.38 ± 0.09 (0.21 to 0.56)	2.88 ± 0.13 (2.62 to 3.14)	<.001
Treatment-by-time = 0.745	TR4	6	5	3.28 ± 0.10 (3.08 to 3.49)	0.33 ± 0.12 (0.10 to 0.56)	2.95 ± 0.16 (2.64 to 3.26)	<.001
	TR5	1	1	3.38 ± 0.21 (2.97 to 3.79)	0.29 ± 0.21 (-0.12 to 0.71)	3.08 ± 0.29 (2.50 to 3.67)	<.001
	TR6	0	1	-	-1.26 ± 0.24 (-1.75 to -0.77)	-	NA
	T1	22	21	3.41 ± 0.07 (3.28 to 3.54)	0.33 ± 0.06 (0.21 to 0.46)	3.08 ± 0.09 (2.90 to 3.26)	<.001
	T2	0	0	-	-	-	NA
Respiratory flow (l s^{-1})	T0[a]	17	19	0.44 ± 0.17 (0.35 to 0.53)	0.44 ± 0.17) (0.35 to 0.52)		
Baseline = 0.731	TR1	19	19	0.62 ± 0.10 (0.42 to 0.83)	0.33 ± 0.10) (0.14 to 0.52)	0.29 ± 0.14 (0.01 to 0.57)	0.041
Treatment = 0.082	TR2	17	19	0.66 ± 0.11 (0.45 to 0.87)	0.45 ± 0.10) (0.26 to 0.64)	0.21 ± 0.14 (-0.07 to 0.50)	0.145
Time = 0.634	TR3	5	6	0.67 ± 0.17 (0.34 to 1.01)	0.26 ± 0.15 (-0.04 to 0.57)	0.41 ± 0.23 (-0.05 to 0.87)	0.077
Treatment-by-time = 0.557	TR4	5	4	0.60 ± 0.18 (0.24 to 0.97)	0.24 ± 0.20 (-0.15 to 0.63)	0.36 ± 0.27 (-0.17) to 0.90	0.181
	TR5	1	1	0.59 ± 0.37 (-0.15 to 1.33)	0.37 ± 0.37 (-0.37) to 1.12)	0.22 ± 0.53 (-0.84) to 1.28)	0.68
	TR6	0	1	-	0.36 ± 0.41 (-0.46) to 1.18)	-	NA
	T1	19	19	0.42 ± 0.10 (0.21 to 0.62)	0.35 ± 0.10 (0.16 to 0.55)	0.06 ± 0.14 (-0.22) to 0.34]	0.668
	T2	0	0	-	-	-	NA

Supplemental Table 1.B: Airway pressure and respiratory flow recordings. Mixed Models for Repeated Measures (MMRM). Per-protocol population.

Variable	Visit	Subjects		Observed values		Treatment differences Adjusted mean (SD) (95%CI)	*P*-value
		CPAP+4 N=17	Sham-CPAP N=20	CPAP+4 Mean (SD) (95%CI)	Sham-CPAP Mean (SD) (95%CI)		
Airway pressure (cm H_2O)	T0[a]	14	20	0.25 ± 0.26 (0.10 to 0.40)	0.30 ± 0.33 (0.14 to 0.46)		
Baseline = 0.009	TR1	17	20	3.29 ± 0.07 (3.15 to 3.44)	0.34 ± 0.06 (0.22 to 0.46)	2.95 ± 0.10 (2.76 to 3.14)	<.001
Treatment = <.001	TR2	17	20	3.29 ± 0.07 (3.14 to 3.43)	0.34 ± 0.06 (0.22 to 0.46)	2.95 ± 0.10 (2.76 to 3.14)	<.001
Time= <.001	TR3	5	6	3.20 ± 0.10 (3.00 to 3.41)	0.39 ± 0.09 (0.21 to 0.57)	2.82 ± 0.14 (2.55 to 3.09)	<.001
Treatment-by-time = 0.681	TR4	5	4	3.22 ± 0.11 (3.00 to 3.44)	0.3 ± 0.1 (0.1 to 0.6)	2.89 ± 0.16 (2.57 to 3.21)	<.001
	TR5	1	1	3.3 ± 0.2 (2.9 to 3.8)	0.33 ± 0.12 (0.10 to 0.56)	3.05 ± 0.30 (2.45 to 3.65)	<.001
	TR6	0	1	-	-1.26 ± 0.25 (-1.75 to -0.77)	-	NA
	T1	17	230	3.37 ± 0.07 (3.23 to 3.52)	0.34 ± 0.06 (0.21 to 0.46)	3.04 ± 0.10 (2.85 to 3.23)	<.001
	T2	0	0	-	-	-	NA
Respiratory flow (l s^{-1})	T0[a]	15	19	0.43 ± 0.17 (0.34 to 0.52)	0.44 ± 0.17 (0.35 to 0.52)		
Baseline = 0.541	TR1	17	19	0.68 ± 0.11 (0.46 to 0.90)	0.33 ± 0.10 (0.14 to 0.52)	0.35 ± 0.15 (0.06 to 0.64)	0.019
Treatment = 0.066	TR2	17	19	0.69 ± 0.11 (0.47 to 0.91)	0.45 ± 0.10 (0.26 to 0.64)	0.24 ± 0.15 (-0.05 to 0.53	0.101
Time = 0.622	TR3	5	6	0.69 ± 0.17 (0.35 to 1.03)	0.26 ± 0.16 (-0.05 to 0.57)	0.42 ± 0.23 (-0.04 to 0.88)	0.070
Treatment-by-time = 0.510	TR4	5	4	0.61 ± 0.18 (0.24 to 0.97)	0.24 ± 0.20 (-0.15 to 0.63)	0.37 ± 0.27 (-0.17 to 0.90)	0.180
	TR5	1	1	0.59 ± 0.37 (-0.16) to 1.33)	0.39 ± 0.38 (-0.36 to 1.13)	0.20 ± 0.53 (-0.86 to 1.26)	0.708
	TR6	0	1	-	0.38 ± 0.41 (-0.45 to 1.20)	-	NA
	T1	17	19	0.44 ± 0.11 (0.23 to 0.66)	0.35 ± 0.10 (0.16 to 0.55)	0.09 ± 0.15 (-0.20 to 0.38)	0.546
	T2	0	0	-	-	-	NA

CPAP+4, continuous positive airway pressure of 4 cm H_2O; ITT, intention-to-treat; NA, not applicable; Sham-CPAP, modified mask for placebo CPAP; T0: baseline; TR (1-6): each end of the ablation cycles (roll-offs); T1: end of RFA, RF electrode inserted, procedural sedation and analgesia (PSA) in progress, patient in RFA position; T2: RFA completed, RF electrode extracted, PSA shutdown, CPAP or Sham device removed, patient in supine position. Treatment refers to either CPAP+4 or Sham-CPAP.

MMRM model definition: Baseline, differences at baseline. If *P* <0.05, means that the groups were different at baseline, and data are adjusted by the baseline value; Treatment, differences between groups regardless of registration times; Time, differences between registration times regardless of treatments; Treatment-by-time, differences between treatments over time.

(a) Baseline airway pressure and airflow were measured with the mask in place.

Supplemental Table 2.1.A. Patient positioning for pulmonary RFA. Categorical variables. Intention-to-treat population.

Variable	Category	CPAP+4 (n=22) n (%)	Sham-CPAP (n=24) n (%)	Total (n=46) n (%)	*P*-value
Treatment position	Supine	8 (36.4%)	7 (29.2%)	15 (32.6%)	0.617
	Prone	12 (54.5%)	14 (58.3%)	26 (56.5%)	
	Left lateral decubitus	0 (0%)	2 (8.3%)	2 (4.3%)	
	Right lateral decubitus	2 (9.1%)	1 (4.2%)	3 (6.5%)	
	Total	22 (100%)	24 (100%)	46 (100%)	

Supplemental Table 2.1.B. Patient positioning for pulmonary RFA. Categorical variables. Per-protocol population.

Variable	Category	CPAP+4 (n=17) n (%)	Sham-CPAP (n=20) n (%)	Total (n=37) n (%)	*P*-value
Treatment position	Supine	6 (35.2%)	7 (35%)	13 (35.1%)	0.940
	Prone	9 (52.9%)	11 (55%)	20 (54.05%)	
	Left lateral decubitus	0 (0%)	1 (5%)	1 (2.7%)	
	Right lateral decubitus	2 (11.7%)	1 (5%)	3 (8.1%)	
	Total	17 (100%)	20 (100%)	37 (100%)	

Supplemental Table 2.2.A. Pulmonary RFA duration. Continuous variables. Intention-to-treat population.

Variable	N CPAP+4	CPAP+4 n=22 Mean ± SD	N Sham-CPAP	Sham-CPAP n=24 Mean ± SD	Total N=46 Mean ± SD	*P*-value
Duration of procedure (min)	22	34.91 ± 23.90	23	42.26 ± 15.93	38.67 ± 20.33	0.229

Supplemental Table 2.2.B Pulmonary RFA duration. Continuous variables. Per-protocol population.

Variable	N CPAP+4	CPAP+4 n=17 Mean ± SD	N Sham-CPAP	Sham-CPAP n=20 Mean ± SD	Total N=37 Mean ± SD	*P*-value
Duration of procedure (min)	17	36.82 ± 24.63	20	41.20 ± 16.55)	39.19 ± 20.47	0.524

Supplemental Table 2.3.A. Anaesthetic drugs. Continuous variables. Intention-to-treat population.

Variable	N CPAP+4	CPAP+4 n=22 Mean ± SD	N Sham-CPAP	Sham-CPAP n=24 Mean ± SD	Total n=46 Mean ± SD	*P*-value
TCI remifentanil (medium dose) (ng ml^{-1})	21	2.38 ± 0.67	23	2.45 ± 0.81	2.42 ± 0.74	0.753
TCI remifentanil (maximum dose) (ng ml^{-1})	21	2.71 ± 0.68	23	2.83 ± 0.76	2.77 ± 0.72	0.611
Total remifentanil dose (µg)	22	271.63 ± 138.09	24	261.77 ± 119.00	266.48 ± 127.13	0.796
Propofol dose (mg)	22	10.40 ± 29.17	23	21.91 ± 39.64	16.28 ± 35.01	0.274
Ketamine dose (mg)	22	5.23 ± 7.32	23	4.35 ± 7.88	4.78 ± 7.53	0.700

Supplementary Table 2.3.B. Anaesthetic drugs. Continuous variables. Per-protocol population.

Variable	N CPAP+4	CPAP+4 n=17 Mean ± SD	N Sham-CPAP	Sham-CPAP n=20 Mean ± SD	Total n=37 Mean ± SD	*P*-value
TCI remifentanil (medium dose) (ng ml^{-1})	17	2.39 ± 0.52	20	2.55 ± 0.82	2.47 ± 0.69	0.501
TCI remifentanil (maximum dose) (ng ml^{-1})	17	2.68 ± 0.55	20	2.87 ± 0.80	2.78 ± 0.70	0.419
Total remifentanil dose (µg)	17	270.82 ± 136.23	20	250.90 ± 103.88	260.05 ± 118.51	0.617
Propofol dose (mg)	17	12.86 ± 32.91	20	23.70 ± 41.98	18.72 ± 37.97	0.394
Ketamine dose (mg)	17	4.71 ± 7.17	20	3.00 ± 6.57	3.78 ± 6.81	0.455

Supplemental Table 2.4.A. Radiofrequency parameters. Continuous variables. Intention-to-treat population.

Variable	N CPAP+4	CPAP+4 n=22 Mean ± SD	N Sham-CPAP	Sham-CPAP n=24 Mean ± SD	Total N=46 Mean ± SD	*P*-value
Maximum generator power (W)	22	66.45 ± 31.34	23	101.61 ± 52.29	84.42 ± 46.38	0.009
Generator impedance (Ohm)	20	548.45 ± 349.35	23	616.61 ± 336.76	584.91 ± 340.29	0.518
Generator thermoablation time (s)	22	732.36 ± 462.09	23	977.04 ± 615.86	857.42 ± 553.94	0.140

Supplemental Table 2.4.B Radiofrequency parameters. Continuous variables. Per-protocol population.

Variable	N CPAP+4	CPAP+4 n=17 Mean ± SD	N Sham-CPAP	Sham-CPAP n=20 Mean ± SD	Total N=37 Mean ± SD	*P*-value
Maximum generator power (W)	17	73.06 ± 29.63	20	97.35 ± 48.10	86.19 ± 41.98	0.079
Generator impedance (Ohm)	17	526.35 ± 345.65	20	626.85 ± 339.48	580.68 ± 341.33	0.379
Generator thermoablation time (s)	17	758.88 ± 462.18	20	846.65 ± 484.96	806.32 ± 470.14	0.578

Supplemental Table 2.5.A: Respiratory monitoring. Mixed Models for Repeated Measures (MMRM). Intention-to-treat population.

Variable	Visit	CPAP+4 N=22	Sham-CPAP N=24	CPAP+4 Mean ± SD (95%CI)	Sham-CPAP Mean ± SD (95%CI)	Treatment differences Adjusted mean ± SD (95%CI)	*P*-value
Respiratory rate (breaths min $^{-1}$)	T0	19	21	19.68 ± 4.75 (17.39 to 21.97)	17.19 ± 6.01 (14.46 to 19.92)		
	TR1	22	22	14.53 ± 0.99 (12.56 to 16.49)	15.74 ± 0.96 (13.84 to 17.64)	-1.21 ± 1.39 (-3.96 to 1.54)	0.384
Baseline = <.001	TR2	20	23	13.07 ± 1.04 (11.01 to 15.12)	16.24 ± 0.94 (14.37 to 18.11)	-3.18 ± 1.41 (-5.97 to -0.39)	0.026
Treatment = 0.716							
Time = <.001	TR3	5	9	15.77 ± 1.77 (12.27 to 19.28)	13.43 ± 1.52 (10.42 to 16.44)	2.34 ± 2.34 (-2.28 to 6.96)	0.318
Treatment-by-time = 0.053							
	TR4	6	7	17.35 ± 1.74 (13.90 to 20.80)	16.79 ± 1.86 (13.11 to 20.46)	0.56 ± 2.54 (-4.47 to 5.59)	0.826
	TR5	1	1	17.41 ± 3.95 (9.59 to 25.23)	14.92 ± 3.96 (7.08 to 22.75)	2.50 ± 5.60 (-8.57 to 13.57)	0.656
	TR6	0	1	-	10.54 ± 4.24 (2.14 to 18.93)	-	NA
	T1	22	23	12.26 ± 0.99 (10.30 to 14.23)	16.01 ± 0.94 (14.14 to 17.87)	-3.74 ± 1.38 (-6.47 to -1.01)	0.008
	T2	20	23	17.95 ± 1.01 (15.94 to 19.95)	18.39 ± 0.94 (16.52 to 20.26)	-0.44 ± 1.39 (-3.19 to 2.32)	0.753
$ETCO_2$ (kPa)	T0	19	21	4.5 ± 0.5 (4.2 to 4.8)	4.4 ± 0.5 (4.2 to 4.7)		
	TR1	22	21	4.7 ± 0.1 (4.3 to 5.1)	4.5 ± 0.1 (4.2 to 4.9)	0.1 ± 0.2 (-0.3 to 0.7)	0.487
Baseline = 0.001	TR2	19	21	4.6 ± 0.2 (4.2 to 5.0)	4.6 ± 0.1 (4.2 to 4.9)	-0.001 ± 0.2 (-0.5 to 0.5)	0.996
Treatment = 0.805							
Time = 0.711	TR3	5	8	4.1 ± 0.3 (3.5 to 4.8)	5.1 ± 0.2 (4.5 to 5.7)	-0.9 ± 0.4 (-1.8 to -0.1)	0.027
Treatment-by-time= 0.143							
	TR4	6	5	4.1 ± 0.3 (3.5 to 4.8)	4.5 ± 0.3 (3.8 to 5.2)	-0.3 ± 0.4 (-1.3 to 0.6)	0.483
	TR5	1	1	4.8 ± 0.7 (3.4 to 6.3)	4.0 ± 0.7 (2.6 to 5.4)	0,8 ± 1.0 (-1.2 to 2.8)	0.417
	TR6	0	1	-	3.6 ± 0.8 (1.9 to 5.2)	-	NA
	T1	21	21	4.6 ± 0.1 (4.2 to 5.0)	4.8 ± 0.1 (4.4 to 5.1)	-0.1 ± 0.2 (-0.7 to 0.3)	0.49
	T2	21	23	4.6 ± 0.1 (4.2 to 5.0)	4.6 ± 0.1 (4.2 to 4.9)	0.03 ± 0.2 (-0.4 to 0.5)	0.89
SpO_2 (%)	T0	20	22	96.70 ± 3.89 (94.88 to 98.52)	97.09 ± 1.87 (96.26 to 97.92)		
	TR1	21	23	97.27 ± 0.57 (96.13 to 98.40)	97.95 ± 0.54 (96.88 to 99.01)	-0.68 ± 0.79 (-2.23 to 0.88)	0.391
Baseline = 0.338							
Treatment = 0.819	TR2	20	23	98.10 ± 0.59 (96.93 to 99.26)	97.99 ± 0.54 (96.93 to 99.06)	0.10 ± 0.80 (-1.47 to 1.68)	0.896
Time = 0.732							
Treatment-by-time= 0.965	TR3	6	9	97.62 ± 0.94 (95.75 to 99.49)	97.03 ± 0.82 (95.40 to 98.66)	0.59 ± 1.25 (-1.89 to 3.07)	0.639
	TR4	6	7	97.63 ± 1.01 (95.63 to 99.63)	97.30 ± 0.99 (95.34 to 99.25)	0.33 ± 1.41 (-2.46 to 3.12)	0.813
	TR5	1	1	97.24 ± 2.26 (92.78 to 101.71)	97.61 ± 2.26 (93.15 to 102.08)	-0.37 ± 3.19 (-6.68 to 5.94)	0.908
	TR6	0	1	-	97.77 ± 2.46 (92.90 to 102.64)	-	NA
	T1	22	23	97.48 ± 0.56 (96.36 to 98.59)	97.99 ± 0.54 (96.93 to 99.06)	-0.52 ± 0.78 (-2.06 to 1.03)	0.509
	T2	21	22	96.88 ± 0.56 (95.76 to 97.99)	97.49 ± 0.54 (96.43 to 98.56)	-0.62 ± 0.78 (-2.16 to 0.93)	0.431

Supplemental Table 2.5.B: Respiratory monitoring. Mixed Models for Repeated Measures (MMRM). Per-protocol population.

Variable	Visit	CPAP+4 N=17	Sham-CPAP N=20	CPAP+4 Mean ± SD (95%CI)	Sham-CPAP Mean ± SD (95%CI)	Treatment differences Adjusted mean ± SD (95%CI)	*P*-value
Respiratory rate (breaths min $^{-1}$)	T0	16	20	18.88 ± 4.66 (16.39 to 21.36)	16.80 ± 5.88 (14.05 to 19.55)		
	TR1	17	19	14.70 ± 1.04 (12.64 to 16.75)	15.35 ± 0.94 (13.48 to 17.22)	-0.66 ± 1.41 (-3.45 to 2.13)	0.642
Baseline = <.001	TR2	17	20	12.57 ± 1.04 (10.52 to 14.62)	15.89 ± 0.93 (14.05 to 17.73)	-3.32 ± 1.40 (-6.08 to -0.56)	0.019
Treatment = 0.972							
Time= <.001	TR3	4	6	17.44 ± 1.92 (13.64 to 21.24)	14.14 ± 1.59 (10.99 to 17.29)	3.30 ± 2.49 (-1.64 to 8.23)	0.188
Treatment-by-time = 0.019							
	TR4	5	4	18.51 ± 1.84 (14.88 to 22.15)	16.59 ± 2.00 (12.63 to 20.55)	1.92 ± 2.71 (-3.45 to 7.29)	0.480
	TR5	1	1	17.87 ± 3.87 (10.21 to 25.53)	14.52 ± 3.88 (6.83 to 22.20)	3.35 ± 5.48 (-7.50 to 14.20)	0.542
	TR6	0	1	-	10.09 ± 4.09 (1.99 to 18.20)	-	NA
	T1	17	20	11.88 ± 1.04 (9.83 to 13.94)	15.74 ± 0.93 (13.90 to 17.58)	-3.86 ± 1.40 (-6.62 to -1.09)	0.007
	T2	16	20	17.65 ± 1.06 (15.55 to 19.76)	18.09 ± 0.93 (16.25 to 19.93)	-0.44 ± 1.42 (-3.24 to 2.37)	0.758
$ETCO_2$ (Kpa)	T0	16	20	4.4 ± 0.5 (4.1 to 4.7)	4.4 ± 0.5 (4.2 to 4.7)		
Baseline = 0.004	TR1	17	20	4.6 ± 0.2 (4.2 to 5.0)	4.6 ± 0.1 (4.2 to 5.0)	0.01 ± 0.2 (-0.5 to 0.5)	0.949
Treatment = 0.395	TR2	17	20	4.5 ± 0.2 (4.1 to 4.9)	4.6 ± 0.1 (4.2 to 5.0)	-0.09 ± 0.2 (-0.6 to 0.4)	0.741
Time= 0.865							
Treatment-by-time = 0.075	TR3	4	6	4.0 ± 0.3 (3.3 to 4.7)	5.0 ± 0.2 (4.4 to 5.6)	-0.9 ± 0.4 (-1.8 to -0.06)	0.036
	TR4	5	4	3.9 ± 0.3 (3.2 to 4.6)	4.7 ± 0.3 (3.9 to 5.5)	-0.8 ± 0.5 (-1.8 to 0.2)	0.121
	TR5	1	1	4.7 ± 0.7 (3.2 to 6.1)	4.1 ± 0.7 (2.7 to 5.5)	0.5 ± 1.0 (-1.4 to 2.5)	0.605
	TR6	0	1	-	3.6 ± 0.7 (2.0 to 5.2]	-	NA
	T1	17	20	4.4 ± 0.2 (4.0 to 4.9)	4,8 ± 0.1 (4.5 to 5.2)	-0.3 ± 0.2 (-0.9 to 0.1)	0.176
	T2	17	20	4.7 ± 0.2 (4.3 to 5.1)	4.5 ± 0.1 (4.2 to 4.9)	0.1 ± 0.2 (-0.4 to 0.7)	0.592
SpO_2 (%)	T0	16	20	97.00 ± 3.83 (94.96 to 99.04)	97.05 ± 1.96 (96.13 to 97.97)		
Baseline = 0.033	TR1	16	20	97.24 ± 0.63 (95.98 to 98.49)	98.28 ± 0.55 (97.19 to 99.38)	-1.05 ± 0.84 (-2.71 to 0.61)	0.213
Treatment = 0.293							
Time = 0.810	TR2	17	20	98.11 ± 0.62 (96.89 to 99.33)	98.28 ± 0.55 (97.19 to 99.38)	-0.18 ± 0.83 (-1.82 to 1.46)	0.831
Treatment-by-time = 0.984							
	TR3	5	6	97.09 ± 1.05 (95.01 to 99.17)	97.74 ± 0.95 (95.85 to 99.63)	-0.65 ± 1.42 (-3.46 to 2.16)	0.648
	TR4	5	4	96.93 ± 1.10 (94.74 to 99.12)	97.88 ± 2.33 (93.28 to 102.49)	-1.03 ± 1.62 (-4.24 to 2.19)	0.527
	TR5	1	1	96.98 ± 2.32 (92.38 to 101.59)	97.88 ± 2.33 (93.28 to 102.49)	-0.90 ± 3.28 (-7.41 to 5.61)	0.785
	TR6	0	1	-	97.86 ± 2.45 (93.01 to 102.70)	-	NA
	T1	17	20	97.42 ± 0.62 (96.20 to 98.64)	98.13 ± 0.55 (97.04 to 99.23)	-0.71 ± 0.83 (-2.35 to 0.92)	0.389
	T2	17	20	96.79 ± 0.62 (95.57 to 98.02)	97.68 ± 0.55 (96.59 to 98.78)	-0.89 ± 0.83 (-2.53 to 0.75)	0.284

Supplemental Table 2.6.A. Temperature monitoring. Mixed Models for Repeated Measures (MMRM). Intention-to-treat population.

Variable	Visit	CPAP+4 N=22	Sham-CPAP N=24	CPAP+4 Mean ± SD (95%CI)	Sham-CPAP Mean ± SD (95%CI)	Treatment differences Adjusted mean ± SD (95%CI)	*P*-value
Tympanic temperature (°C)	T0	22	24	35.65 ± 0.71 (35.34 to 35.97)	35.69 ± 0.54 (35.47 to 35.92)		
Baseline = <.001	TR 1	22	23	35.83 ± 0.12 (35.59 to 36.06)	35.77 ± 0.12 (35.54 to 36.00)	0.06 ± 0.17 (-0.27 to 0.39)	0.713
Treatment = 0.987	TR 2	20	23	35.75 ± 0.12 (35.51 to 35.99)	35.72 ± 0.12 (35.49 to 35.95)	0.03 ± 0.17 (-0.30 to 0.36)	0.862
Time = 0.012							
Treatment-by-time = 0.098	TR 3	6	9	35.76 ± 0.15 (35.47 to 36.05)	35.63 ± 0.13 (35.37 to 35.89)	0.13 ± 0.20 (-0.26 to 0.53)	0.509
	TR 4	6	7	35.64 ± 0.17 (35.31 to 35.97)	35.79 ± 0.15 (35.49 to 36.09)	-0.15 ± 0.23 (-0.60 to 0.29)	0.494
	TR 5	1	1	35.42 ± 0.29 (34.85 to 35.99)	35.71 ± 0.28 (35.16 to 36.27)	-0.30 ± 0.40 (-1.10 to 0.50)	0.464
	TR 6	0	1	-	35.64 ± 0.35 (34.94 to 36.34)	-	NA
	T1	21	23	35.74 ± 0.12 (35.50 to 35.97)	35.72 ± 0.12 (35.49 to 35.95)	0.02 ± 0.17 (-0.31 to 0.35)	0.926
	T2	22	23	35.70 ± 0.12 (35.46 to 35.93)	35.50 ± 0.12 (35.27 to 35.73)	0.19 ± 0.17 (-0.13 to 0.52)	0.245

Supplemental Table 2.6.B. Temperature monitoring. Mixed Models for Repeated Measures (MMRM). Per-protocol population.

Variable	Visit	CPAP+4 N=17	Sham-CPAP N=20	CPAP+4 Mean ± SD (95%CI)	Sham-CPAP Mean ± SD [95%CI]	Treatment differences Adjusted mean ± SD (95%CI)	*P*-value
Tympanic temperature (°C)	T0	17	20	35.56 ± 0.78 (35.16 to 35.97)	35.63 ± 0.49) (35.40 to 35.86)		
Baseline = <.001	TR 1	17	20	35.76 ± 0.12 (35.53 to 36.00)	35.68 ± 0.11) (35.46 to 35.89)	0.08 ± 0.16 (-0.23 to 0.40)	0.602
Treatment = 0.459	TR 2	17	20	35.75 ± 0.12 (35.52 to 35.99)	35.62 ± 0.11) (35.41 to 35.84)	0.13 ± 0.16 (-0.19 to 0.45)	0.431
Time = 0.010							
Treatment-by-time = 0.083	TR 3	5	6	35.73 ± 0.14 (35.45 to 36.02)	35.47 ± 0.13) (35.21 to 35.73)	0.27 ± 0.19 (-0.12 to 0.65)	0.171
	TR 4	5	4	35.58 ± 0.16 (35.26 to 35.89)	35.51 ± 0.16) (35.20 to 35.83)	0.06 ± 0.23 (-0.39 to 0.51)	0.784
	TR 5	1	1	35.35 ± 0.26 (34.84 to 35.87)	35.46 ± 0.26) (34.94 to 35.97)	-0.10 ± 0.37 (-0.83 to 0.62)	0.777
	TR 6	0	1	-	35.41 ± 0.32) (34.78 to 36.03)	-	NA
	T1	17	20	35.67 ± 0.12 (35.43 to 35.90)	35.57 ± 0.11) (35.36 to 35.79)	0.10 ± 0.16 (-0.22 to 0.41)	0.556
	T2	17	20	35.67 ± 0.12 (35.43 to 35.90)	35.37 ± 0.11) (35.15 to 35.58)	0.30 ± 0.16 (-0.02 to 0.62)	0.065

Supplemental Table 2.7.A. Ramsay sedation scale. Intention-to-treat population.

Time	Ramsay	CPAP+4 (n=22) n (%)	Sham-CPAP (n=24) n (%)	Total (n=46) n (%)	P- value
T0	1	0 (0%)	1 (4.2%)	1 (2.2%)	0.863
	2	16 (72.7%)	18 (75%)	34 (73.9%)	
	3	6 (27.3%)	5 (20.8%)	11 (23.9%)	
	Total	22 (100%)	24 (100%)	46 (100%)	
TR1	2	0 (0%)	5 (21.7%)	5 (11.1%)	0.024
	3	20 (90.9%)	18 (78.3%)	38 (84.4%)	
	4	2 (9.1%)	0 (0%)	2 (4.4%)	
	Total	22 (100%)	23 (100%)	45 (100%)	
TR2	2	2 (10%)	5 (21.7%)	7 (16.3%)	0.552
	3	14 (70%)	16 (69.6%)	30 (69.8%)	
	4	3 (15%)	2 (8.7%)	5 (11.6%)	
	5	1 (5%)	0 (0%)	1 (2.3%)	
	Total	20 (100%)	23 (100%)	43 (100%)	
TR3	2	1 (16.7%)	2 (22.2%)	3 (20%)	1.000
	3	4 (66.7%)	6 (66.7%)	10 (66.7%)	
	4	1 (16.7%)	1 (11.1%)	2 (13.3%)	
	Total	6 (100%)	9 (100%)	15 (100%)	
TR4	2	0 (0%)	1 (14.3%)	1 (7.7%)	1.000
	3	5 (83.3%)	6 (85.7%)	11 (84.6%)	
	4	1 (16.7%)	0 (0%)	1 (7.7%)	
	Total	6 (100%)	7 (100%)	13 (100%)	
TR5	3	1 (100%)	1 (100%)	2 (100%)	NA
	Total	1 (100%)	1 (100%)	2 (100%)	
TR6	3	0 (0%)	1 (100%)	1 (100%)	NA
	Total	0 (0%)	1 (100%)	1 (100%)	
T1	2	1 (4.5%)	2 (8.7%)	3 (6.7%)	1.000
	3	18 (81.8%)	19 (82.6%)	37 (82.2%)	
	4	3 (13.6%)	2 (8.7%)	5 (11.1%)	
	Total	22 (100%)	23 (100%)	45 (100%)	
T2	2	14 (63.6%)	20 (87.0%)	34 (75.6%)	0.090
	3	8 (36.4%)	3 (13.0%)	11 (24.4%)	
	Total	22 (100%)	23 (100%)	45 (100%)	

Supplemental Table 2.7.B. Ramsay sedation scale. Per-protocol population.

Time	Ramsay	CPAP+4 (n=17) n (%)	Sham-CPAP (n=20) n (%)	Total (n=37) n (%)	*P*- value
T0	1	0 (0%)	1 (5%)	1 (2.7%)	1.000
	2	13 (76.4%)	14 (70%)	27 (72.9%)	
	3	4 (23.5%)	5 (25%)	9 (24.3%)	
	Total	17 (100%)	20 (100%)	37 (100%)	
TR1	2	0 (0%)	4 (20%)	4 (10.8%)	0.110
	3	16 (94.1%)	16 (80%)	32 (86.4%)	
	4	1 (5.8%)	0 (0%)	1 (2.7%)	
	Total	17 (100%)	20 (100%)	37 (100%)	
TR2	2	2 (11.7%)	4 (20%)	6 (16.2%)	0.869
	3	13 (76.4%)	14 (70%)	27 (72.9%)	
	4	2 (11.7%)	2 (10%)	4 (10.8%)	
	Total	17 (100%)	20 (100%)	37 (100%)	
	2	1 (20%)	2 (33.3%)	3 (27.2%)	1.000
TR3	3	4 (80%)	4 (66.6%)	8 (72.7%)	
	Total	4 (80%)	4 (66.7%)	8 (72.7%)	
	2	0 (0%)	1 (25%)	1 (11.1%)	0.444
TR4	3	5 (100%)	3 (75%)	8 (88.8%)	
	Total	5 (100%)	4 (100%)	9 (100%)	
TR5	3	1 (100%)	1 (100%)	2 (100%)	NA
	Total	1 (100%)	1 (100%)	2 (100%)	
TR6	3	0 (0%)	1 (100%)	1 (100%)	NA
	Total	0 (0%)	1 (100%)	1 (100%)	
T1	2	1 (5.8%)	2 (10%)	3 (8.1%)	1.000
	3	15 (88.2%)	16 (80%)	31 (83.7%)	
	4	1 (5.8%)	2 (10%)	3 (8.1%)	
	Total	17 (100%)	20 (100%)	37 (100%)	
T2	2	10 (58.8%)	17 (85%)	27 (72.9%)	0.134
	3	7 (41.1%)	3 (15%)	10 (27.03%)	
	Total	17 (100%)	20 (100%)	37 (100%)	

Supplemental Table 2.8.A. BIS monitoring. Intention-to-treat population.

Variable	Visit	N CPAP	CPAP+4 n=22 Mean ± SD	N Sham-CPAP	Sham-CPAP n=24 Mean ± SD	Total N=46 Mean ± SD	*P*- value
BIS	T0	22	92.86 ± 5.37	24	93.63 ± 5.05	93.26 ± 5.17	0.632
	TR1	22	88.27 ± 9.13	23	85.22 ± 10.58	86.71 ± 9.91	0.306
	TR2	20	87.95 ± 7.47	23	84.70 ± 10.42	86.21 ± 9.21	0.252
	TR3	6	88.33 ± 7.58	9	88.89 ± 9.31	88.67 ± 8.37	0.905
	TR4	6	90.83 ± 7.14	7	88.57 ± 8.06	89.62 ± 7.42	0.255
	TR5	1	97	1	80	88.5	NA
	TR6	0	-	1	80	80	NA
	T1	21	89.10 ± 7.42	23	85.78 ± 11.02	87.36 ± 9.52	0.253
	T2	22	91.45 ± 6.19	23	94.30 ± 4.15	92.91 ± 5.38	0.075

Supplemental Table 2.8.B. BIS monitoring. Per-protocol population.

Variable	Visit	N CPAP	CPAP+4 n=17 Mean ± SD	N Sham-CPAP	Sham-CPAP n=20 Mean ± SD	Total N=37 Mean ± SD	*P*- value
BIS	T0	17	93.41 ± 4.37	20	93.50 ± 5.07	93.46 ± 4.70	0.955
	TR1	17	89.47 ± 7.83	20	84.50 ± 10.97	86.78 ± 9.85	0.127
	TR2	17	88.06 ± 7.61	20	84.30 ± 11.09	86.03 ± 9.71	0.245
	TR3	5	90.20 ± 6.76	6	89.50 ± 9.25	89.82 ± 7.82	0.891
	TR4	5	93.40 ± 3.78	4	93.75 ± 2.99	93.56 ± 3.24	0.884
	TR5	1	97	1	80	88.50 ± 12.02	NA
	TR6	0	-	1	80	80	NA
	T1	16	89.31 ± 7.70	20	85.85 ± 11.20	87.39 ± 9.83	0.300
	T2	17	92.18 ± 6.52	20	94.50 ± 4.27	93.43 ± 5.47	0.202

Supplemental Table 2.9.A. BIS monitoring. Mixed Models for Repeated Measures (MMRM). Per-protocol population.

Variable	Visit	CPAP+4 N=22	Sham-CPAP N=24	CPAP+4 Mean ± SD (95%CI)	Sham-CPAP Mean ± SD (95%CI)	Treatment differences Adjusted mean ± SD (95%CI)	*P*- value
BIS	T0	22	24	92.86 ± 5.37 (90.48 to 95.25)	93.63 ± 5.05 (91.49 to 95.76)		
Baseline = 0.174	TR1	22	23	88.33 ± 1.79 (84.80 to 91.86)	85.11 ± 1.75 (81.66 to 88.57)	3.22 ± 2.50 (-1.73 to 8.16)	0.201
Treatment = 0.034<br>Time = <.001	TR2	20	23	88.17 ± 1.85 (84.52 to 91.83)	84.59 ± 1.75 (81.14 to 88.05)	3.58 ± 2.55 (-1.45 to 8.61)	0.162
Treatment-By-time = 0.063	TR3	6	9	89.40 ± 3.07 (83.34 to 95.46)	88.12 ± 2.54 (83.10 to 93.15)	1.28 ± 3.99 (-6.61 to 9.17)	0.749
	TR4	6	7	91.76 ± 3.34 (85.15 to 98.37)	87.92 ± 3.00 (81.98 to 93.85)	3.84 ± 4.50 (5.05 to 12.74)	0.395
	TR5	1	1	98.92 ± 7.30 (84.50 to 113.34)	75.68 ± 7.27 (61.32 to 90.04)	23.24 ± 10.31 (2.88 to 43.60)	0.026
	TR6	0	1	-	77.16 ± 8.10 (61.16 to 93.16)	-	NA
	T1	21	23	89.43 ± 1.81 (85.85 to 93.01)	85.68 ± 1.75 (82.22 to 89.14)	3.75 ± 2.52 (-1.23 to 8.73)	0.139
	T2	22	23	91.51 ± 1.79 (87.98 to 95.05)	94.20 ± 1.75 (90.75 to 97.66)	-2.69 ± 2.50 (-7.63 to 2.26)	0.284

Supplemental Table 2.9.B. BIS monitoring. Mixed Models for Repeated Measures (MMRM). Per-protocol population.

Variable	Visit	CPAP+4 N=17	Sham-CPAP N=20	CPAP+4 Mean ± SD (95%CI)	Sham-CPAP Mean ± SD (95%CI)	Treatment differences Adjusted mean ± SD (95%CI)	*P*- value
BIS	T0	17	20	93.41 ± 4.37 (91.16 to 95.66)	93.50 ± 5.07 (91.13 to 95.87)		
Baseline = 0.471	TR1	17	20	89.48 ± 2.04 (85.43 to 93.52)	84.49 ± 1.88 (80.76 to 88.22)	4.98 ± 2.78 (-0.52 to 10.49)	0.075
Treatment = 0.076<br>Time = <.001	TR2	17	20	88.07 ± 2.04 (84.02 to 92.11)	84.29 ± 1.88 (80.56 to 88.02)	3.77 ± 2.78 (-1.73 to 9.28)	0.177
Treatment-By-time = 0.072	TR3	5	6	89.95 ± 3.26 (83.50 to 96.39)	89.01 ± 2.98 (83.11 to 94.90)	0.94 ± 4.41 (-7.80 to 9.68)	0.832
	TR4	5	4	93.32 ± 3.59 (86.20 to 100.43)	92.88 ± 3.81 (85.33 to 100.44)	0.43 ± 5.24 (-9.95 to 10.81)	0.934
	TR5	1	1	100.15 ± 7.08 (86.14 to 114.17)	78.44 ± 7.13 (4.32 to 92.56)	21.72 ± 10.05 (1.82 to 41.61)	0.033
	TR6	0	1	-	78.77 ± 8.01 (62.92 to 94.62)	-	NA
	T1	16	20	89.64 ± 2.07 (85.53 to 93.74)	85.84 ± 1.88 (82.11 to 89.57)	3.80 ± 2.80 (-1.75 to 9.34)	0.178
	T2	17	20	92.18 ± 2.04 (88.14 to 96.23)	94.49 ± 1.88 (90.76 to 98.22)	-2.31 ± 2.78 (-7.81 to 3.19)	0.408

BIS, bispectral index; CPAP+4, continuous positive airway pressure of 4 cm H_2O; $ETCO_2$, end-tidal CO_2; NA, not applicable; RFA, radiofrequency ablation; Sham-CPAP, modified mask for placebo CPAP; SpO_2, oxygen saturation measured by pulse oximetry; T0: baseline; TR (1-6): each end of the ablation cycles (roll-offs); T1: end of RFA, RF electrode inserted, procedural sedation and analgesia (PSA) in progress, patient in RFA position; T2: RFA completed, RF electrode extracted, PSA shutdown, CPAP or Sham device removed, patient in supine position; TCI, target controlled infusion. Treatment refers to either CPAP+4 or Sham-CPAP.

MMRM model definition: Baseline, differences at baseline. If $P < 0.05$, means that the groups were different at baseline, and data are adjusted by the baseline value; Treatment, differences between groups regardless of registration times; Time, differences between registration times regardless of treatments; Treatment-by-time, differences between treatments over time.

Supplemental Table 3.A: Adverse Events in order of frequency. Number (%) of subjects reporting adverse events by Medical Dictionary for Regulatory Activities (MedDRA) Preferred term. Intention-to-treat population.

Preferred Term	CPAP+4 (N=22) n (%)	Sham-CPAP (N=24) n (%)	Total (N=46) n (%)	*P*-value	OR (95%CI)	*P*- value logistic model
Hypercapnia	21 (95.5%)	18 (75.0%)	39 (84.8%)	0.084	7 (0.769 to 63.722)	0.084
Chest pain	18 (81.8%)	18 (75.0%)	36 (78.3%)	0.627	1.421 (0.344 to 5.878)	0.627
Pain in extremity	12 (54.5%)	19 (79.2%)	31 (67.4%)	0.080	0.316 (0.087 to 1.152)	0.080
Traumatic pneumothorax	16 (72.7%)	14 (58.3%)	30 (65.2%)	0.308	1.905 (0.551 to 6.585)	0.308
Hypoxia	13 (59.1%)	15 (62.5%)	28 (60.9%)	0.813	0.867 (0.265 to 2.836)	0.813
Hypopnoea	12 (54.5%)	15 (62.5%)	27 (58.7%)	0.584	0.72 (0.222 to 2.338)	0.584
Pulmonary haemorrhage	9 (40.9%)	13 (54.2%)	22 (47.8%)	0.464	0.651 (0.206 to 2.057)	0.464
Hypertension during therapeutic procedure	13 (59.1%)	8 (33.3%)	21 (45.7%)	0.083	2.889 (0.869 to 9.601)	0.083
Apnoea	10 (45.5%)	7 (29.2%)	17 (37.0%)	0.255	2.024 (0.6 to 6.828)	0.255
Feeling hot	5 (22.7%)	11 (45.8%)	16 (34.8%)	0.105	0.348 (0.097 to 1.250)	0.105
Sweating	2 (9.1%)	12 (50.0%)	14 (30.4%)	0.006	0.1 (0.019 to 0.526)	0.006
Bradypnoea	8 (36.4%)	5 (20.8%)	13 (28.3%)	0.247	2.171 (0.584 to 8.075)	0.247
Subcutaneous emphysema	7 (31.8%)	5 (20.8%)	12 (26.1%)	0.399	1.773 (0.468 to 6.721)	0.399
Haemoptysis	3 (13.6%)	8 (33.3%)	11 (23.9%)	0.216	0.421 (0.107 to 1.661)	0.216
Pleural effusion	5 (22.7%)	4 (16.7%)	9 (19.6%)	0.605	1.471 (0.34 to 6.365)	0.605
Nausea	4 (18.2%)	4 (16.7%)	8 (17.4%)	0.892	1.111 (0.242 to 5.107)	0.892
Asthenia	3 (13.6%)	2 (8.3%)	5 (10.9%)	0.567	1.737 (0.262 to 11.515)	0.567
Dyspnoea	3 (13.6%)	2 (8.3%)	5 (10.9%)	0.567	1.737 (0.262 to 11.515)	0.567
Cough	3 (13.6%)	2 (8.3%)	5 (10.9%)	0.567	1.737 (0.262 to 11.515)	0.567
Vomiting	4 (18.2%)	1 (4.2%)	5 (10.9%)	0.160	5.111 [0.525 to 49.793)	0.160
Diarrhoea	2 (9.1%)	2 (8.3%)	4 (8.7%)	0.927	1.1 (0.141 to 8.556)	0.927
Thoracic cavity drainage	3 (13.6%)	1 (4.2%)	4 (8.7%)	0.280	3.631 (0.349 to 37.814)	0.280
Procedural anxiety	1 (4.5%)	2 (8.3%)	3 (6.5%)	0.608	0.524 (0.044 to 6.216)	0.608
Atelectasis	2 (9.1%)	1 (4.2%)	3 (6.5%)	0.509	2.3 (0.194 to 27.304)	0.509
Bradycardia	0 (0.0%)	3 (12.5%)	3 (6.5%)	0.234	NA	NA
Pleuritic pain	3 (13.6%)	0 (0.0%)	3 (6.5%)	0.101	NA	NA
Haemothorax	2 (9.1%)	1 (4.2%)	3 (6.5%)	0.509	2.3 (0.194 to 27.304)	0.509
Viral respiratory tract infection	1 (4.5%)	2 (8.3%)	3 (6.5%)	0.608	0.524 (0.044 to 6.216)	0.608
Dizziness	2 (9.1%)	1 (4.2%)	3 (6.5%)	0.509	2.300 (0.194 to 27.304)	0.509
Anaemia	1 (4.5%)	1 (4.2%)	2 (4.3%)	0.949	1.095 (0.064 to 18.638)	0.949
Decreased appetite	1 (4.5%)	1 (4.2%)	2 (4.3%)	0.949	1.095 (0.064 to 18.638)	0.949
Aspiration of pleural cavity	1 (4.5%)	1 (4.2%)	2 (4.3%)	0.949	1.095 (0.064 to 18.638)	0.949
Headache	1 (4.5%)	1 (4.2%)	2 (4.3%)	0.949	1.095 (0.064 to 18.638)	0.949
Musculoskeletal pain	1 (4.5%)	1 (4.2%)	2 (4.3%)	0.949	1.095 (0.064 to 18.638)	0.949
Constipation	2 (9.1%)	0 (0.0%)	2 (4.3%)	0.223	NA	NA

**Preferred Term**	**CPAP+4 (N=22) n (%)**	**Sham-CPAP (N=24) n (%)**	**Total (N=46) n (%)**	***P*- value**	**OR (95%CI)**	***P*- value logistic model**
Device failure	1 (4.5%)	1 (4.2%)	2 (4.3%)	0.949	1.095 (0.064 to 18.638)	0.949
Procedural hypotension	0 (0.0%)	2 (8.3%)	2 (4.3%)	0.489	NA	NA
Upper respiratory tract infection	2 (9.1%)	0 (0.0%)	2 (4.3%)	0.223	NA	NA
Urinary tract infection	2 (9.1%)	0 (0.0%)	2 (4.3%)	0.223	NA	NA
Lung infection	0 (0.0%)	2 (8.3%)	2 (4.3%)	0.489	NA	NA
Postoperative respiratory failure	2 (9.1%)	0 (0.0%)	2 (4.3%)	0.223	NA	NA
Therapy cessation	1 (4.5%)	1 (4.2%)	2 (4.3%)	0.949	1.095 (0.064 to 18.638)	0.949
Laceration	0 (0.0%)	2 (8.3%)	2 (4.3%)	0.489	NA	NA
Feeling cold	2 (9.1%)	0 (0.0%)	2 (4.3%)	0.223	NA	NA
Dysaesthesia	0 (0.0%)	1 (4.2%)	1 (2.2%)	0.477	NA	NA
Skin abrasion	1 (4.5%)	0 (0.0%)	1 (2.2%)	0.478	NA	NA
Anxiety	1 (4.5%)	0 (0.0%)	1 (2.2%)	0.478	NA	NA
Arthralgia	0 (0.0%)	1 (4.2%)	1 (2.2%)	0.477	NA	NA
Arthritis	0 (0.0%)	1 (4.2%)	1 (2.2%)	0.477	NA	NA
Renal colic	0 (0.0%)	1 (4.2%)	1 (2.2%)	0.477	NA	NA
Fall	0 (0.0%)	1 (4.2%)	1 (2.2%)	0.477	NA	NA
Pulmonary cavitation	1 (4.5%)	0 (0.0%)	1 (2.2%)	0.478	NA	NA
Depression	0 (0.0%)	1 (4.2%)	1 (2.2%)	0.477	NA	NA
Dysphagia	0 (0.0%)	1 (4.2%)	1 (2.2%)	0.477	NA	NA
Dysuria	1 (4.5%)	0 (0.0%)	1 (2.2%)	0.478	NA	NA
Back pain	0 (0.0%)	1 (4.2%)	1 (2.2%)	0.477	NA	NA
Intercepted product preparation error	1 (4.5%)	0 (0.0%)	1 (2.2%)	0.478	NA	NA
Muscle spams	1 (4.5%)	0 (0.0%)	1 (2.2%)	0.478	NA	NA
Sneezing	1 (4.5%)	0 (0.0%)	1 (2.2%)	0.478	NA	NA
Ventricular extrasystoles	0 (0.0%)	1 (4.2%)	1 (2.2%)	0.477	NA	NA
Pleural fistula	1 (4.5%)	0 (0.0%)	1 (2.2%)	0.478	NA	NA
Gastritis	0 (0.0%)	1 (4.2%)	1 (2.2%)	0.477	NA	NA
Gastroenteritis	1 (4.5%)	0 (0.0%)	1 (2.2%)	0.478	NA	NA
Skin haemorrhage	0 (0.0%)	1 (4.2%)	1 (2.2%)	0.477	NA	NA
Puncture site haemorrhage	1 (4.5%)	0 (0.0%)	1 (2.2%)	0.478	NA	NA
Hyperglycaemia	0 (0.0%)	1 (4.2%)	1 (2.2%)	0.477	NA	NA
Drug hypersensitivity	1 (4.5%)	0 (0.0%)	1 (2.2%)	0.478	NA	NA
Hypertension	1 (4.5%)	0 (0.0%)	1 (2.2%)	0.478	NA	NA
Tooth infection	0 (0.0%)	1 (4.2%)	1 (2.2%)	0.477	NA	NA
Postural dizziness	0 (0.0%)	1 (4.2%)	1 (2.2%)	0.477	NA	NA
Hepatic metastases	0 (0.0%)	1 (4.2%)	1 (2.2%)	0.477	NA	NA
Migraine	1 (4.5%)	0 (0.0%)	1 (2.2%)	0.478	NA	NA
Orthopnoea	1 (4.5%)	0 (0.0%)	1 (2.2%)	0.478	NA	NA
Pallor	0 (0.0%)	1 (4.2%)	1 (2.2%)	0.477	NA	NA
Paraesthesia	0 (0.0%)	1 (4.2%)	1 (2.2%)	0.477	NA	NA
Weight decreased	1 (4.5%)	0 (0.0%)	1 (2.2%)	0.478	NA	NA
Petechiae	1 (4.5%)	0 (0.0%)	1 (2.2%)	0.478	NA	NA
Adverse drug reaction	0 (0.0%)	1 (4.2%)	1 (2.2%)	0.477	NA	NA
Decreased platelet count	1 (4.5%)	0 (0.0%)	1 (2.2%)	0.478	NA	NA
Retained bronchial secretion	1 (4.5%)	0 (0.0%)	1 (2.2%)	0.478	NA	NA
Allergic cough	1 (4.5%)	0 (0.0%)	1 (2.2%)	0.478	NA	NA
Productive cough	0 (0.0%)	1 (4.2%)	1 (2.2%)	0.477	NA	NA
Cognitive disorder	1 (4.5%)	0 (0.0%)	1 (2.2%)	0.478	NA	NA

Supplemental Table 3.B: Adverse events in order of frequency. Number (%) of subjects reporting adverse events by Medical Dictionary for Regulatory Activities (MedDRA) Preferred term. Per-protocol population.

Preferred Term	CPAP+4 (N=17) n (%)	Sham-CPAP (N=20) n (%)	Total (N=37) n (%)	*P*-value	OR (95%CI)	*P*- value logistic model
Hypercapnia	16 (94.1%)	16 (80.0%)	32 (86.5%)	0.237	3.999 (0.402 to 39.804)	0.237
Chest pain	15 (88.2%)	15 (75.0%)	30 (81.1%)	0.315	2.5 (0.418 to 14.961)	0.315
Pain in extremity	9 (52.9%)	16 (80.0%)	25 (67.6%)	0.086	0.281 (0.066 to 1.201)	0.086
Hypopnoea	10 (58.8%)	15 (75.0%)	25 (67.6%)	0.298	0.476 (0.118 to 1.929)	0.298
Traumatic pneumothorax	13 (76.5%)	12 (60.0%)	25 (67.6%)	0.290	2.167 (0.516 to 9.09)	0.290
Hypoxia	11 (64.7%)	11 (55.0%)	22 (59.5%)	0.549	1.5 (0.397 to 5.664)	0.549
Puncture site haemorrhage	8 (47.1%)	10 (50.0%)	18 (48.6%)	0.858	0.889 (0.244 to 3.243)	0.858
Hypertension during therapeutic procedure	10 (58.8%)	7 (35.0%)	17 (45.9%)	0.151	2.653 (0.699 to 10.062)	0.151
Apnoea	8 (47.1%)	7 (35.0%)	15 (40.5%)	0.457	1.651 (0.44 to 6.2)	0.457
Feeling hot	4 (23.5%)	10 (50.0%)	14 (37.8%)	0.104	0.308 (0.074 to 1.276)	0.104
Sweating	2 (11.8%)	11 (55.0%)	13 (35.1%)	0.011	0.109 (0.02 to 0.608)	0.011
Bradypnoea	7 (41.2%)	5 (25.0%)	12 (32.4%)	0.298	2.1 (0.518 to 8.506)	0.298
Subcutaneous emphysema	7 (41.2%)	5 (25.0%)	12 (32.4%)	0.298	2.1 (0.518 to 8.506)	0.298
Haemoptysis	3 (17.6%)	8 (40.0%)	11 (29.7%)	0.147	0.321 (0.069 to 1.491)	0.147
Pleural effusion	5 (29.4%)	4 (20.0%)	9 (24.3%)	0.508	1.667 (0.367 to 7.566)	0.508
Nausea	2 (11.8%)	3 (15.0%)	5 (13.5%)	0.774	0.756 (0.111 to 5.149)	0.774
Asthenia	2 (11.8%)	2 (10.0%)	4 (10.8%)	0.863	1.2 (0.15 to 9.57)	0.863
Cough	2 (11.8%)	2 (10.0%)	4 (10.8%)	0.863	1.2 (0.15 to 9.57)	0.863
Vomiting	3 (17.6%)	1 (5.0%)	4 (10.8%)	0.244	4.071 (0.382 to 43.368)	0.244
Procedural anxiety	1 (5.9%)	2 (10.0%)	3 (8.1%)	0.651	0.563 (0.046 to 6.806)	0.651
Atelectasis	2 (11.8%)	1 (5.0%)	3 (8.1%)	0.465	2.533 (0.209 to 30.68)	0.465
Bradycardia	0 (0.0%)	3 (15.0%)	3 (8.1%)	0.234	NA	NA
Diarrhoea	1 (5.9%)	2 (10.0%)	3 (8.1%)	0.651	0.563 (0.046 to 6.806)	0.651
Thoracic cavity drainage	3 (17.6%)	0 (0.0%)	3 (8.1%)	0.087	NA	NA
Haemothorax	2 (11.8%)	1 (5.0%)	3 (8.1%)	0.465	2.533 (0.209 to 30.68)	0.465
Viral respiratory tract infection	1 (5.9%)	2 (10.0%)	3 (8.1%)	0.651	0.563 (0.046 to 6.806)	0.651
Decreased appetite	1 (5.9%)	1 (5.0%)	2 (5.4%)	0.905	1.188 (0.069 to 20.539)	0.905
Aspiration of pleural cavity	1 (5.9%)	1 (5.0%)	2 (5.4%)	0.905	1.188 (0.069 to 20.539)	0.905
Dyspnoea	1 (5.9%)	1 (5.0%)	2 (5.4%)	0.905	1.188 (0.069 to 20.539)	0.905
Musculoskeletal pain	1 (5.9%)	1 (5.0%)	2 (5.4%)	0.905	1.188 (0.069 to 20.539)	0.905
Pleuritic pain	2 (11.8%)	0 (0.0%)	2 (5.4%)	0.204	NA	NA
Constipation	2 (11.8%)	0 (0.0%)	2 (5.4%)	0.204	NA	NA
Procedural hypotension	0 (0.0%)	2 (10.0%)	2 (5.4%)	0.489	NA	NA
Lung infection	0 (0.0%)	2 (10.0%)	2 (5.4%)	0.489	NA	NA
Laceration	0 (0.0%)	2 (10.0%)	2 (5.4%)	0.489	NA	NA

Preferred Term	CPAP+4 (N=17) n (%)	Sham-CPAP (N=20) n (%)	Total (N=37) n (%)	*P*-value	OR (95%CI)	*P*- value logistic model
Dizziness	1 (5.9%)	1 (5.0%)	2 (5.4%)	0.905	1.188 (0.069 to 20.539)	0.905
Skin abrasion	1 (5.9%)	0 (0.0%)	1 (2.7%)	0.459	NA	NA
Arthralgia	0 (0.0%)	1 (5.0%)	1 (2.7%)	1.000	NA	NA
Arthritis	0 (0.0%)	1 (5.0%)	1 (2.7%)	1.000	NA	NA
Renal colic	0 (0.0%)	1 (5.0%)	1 (2.7%)	1.000	NA	NA
Fall	0 (0.0%)	1 (5.0%)	1 (2.7%)	1.000	NA	NA
Pulmonary cavitation	1 (5.9%)	0 (0.0%)	1 (2.7%)	0.459	NA	NA
Headache	0 (0.0%)	1 (5.0%)	1 (2.7%)	1.000	NA	NA
Depression	0 (0.0%)	1 (5.0%)	1 (2.7%)	1.000	NA	NA
Dysaesthesia	0 (0.0%)	1 (5.0%)	1 (2.7%)	1.000	NA	NA
Dysphagia	0 (0.0%)	1 (5.0%)	1 (2.7%)	1.000	NA	NA
Dysuria	1 (5.9%)	0 (0.0%)	1 (2.7%)	0.459	NA	NA
Back pain	0 (0.0%)	1 (5.0%)	1 (2.7%)	1.000	NA	NA
Muscle spams	1 (5.9%)	0 (0.0%)	1 (2.7%)	0.459	NA	NA
Ventricular extrasystoles	0 (0.0%)	1 (5.0%)	1 (2.7%)	1.000	NA	NA
Pleural fistula	1 (5.9%)	0 (0.0%)	1 (2.7%)	0.459	NA	NA
Device failure	0 (0.0%)	1 (5.0%)	1 (2.7%)	1.000	NA	NA
Gastritis	0 (0.0%)	1 (5.0%)	1 (2.7%)	1.000	NA	NA
Gastroenteritis	1 (5.9%)	0 (0.0%)	1 (2.7%)	0.459	NA	NA
Skin haemorrhage	1 (5.9%)	0 (0.0%)	1 (2.7%)	0.459	NA	NA
Hyperglycaemia	0 (0.0%)	1 (5.0%)	1 (2.7%)	1.000	NA	NA
Hypertension	1 (5.9%)	0 (0.0%)	1 (2.7%)	0.459	NA	NA
Upper respiratory tract infection	1 (5.9%)	0 (0.0%)	1 (2.7%)	0.459	NA	NA
Tooth infection	0 (0.0%)	1 (5.0%)	1 (2.7%)	1.000	NA	NA
Postoperative respiratory failure	1 (5.9%)	0 (0.0%)	1 (2.7%)	0.459	NA	NA
Therapy cessation	1 (5.9%)	0 (0.0%)	1 (2.7%)	0.459	NA	NA
Postural dizziness	0 (0.0%)	1 (5.0%)	1 (2.7%)	1.000	NA	NA
Hepatic metastases	0 (0.0%)	1 (5.0%)	1 (2.7%)	1.000	NA	NA
Paraesthesia	0 (0.0%)	1 (5.0%)	1 (2.7%)	1.000	NA	NA
Petechiae	1 (5.9%)	0 (0.0%)	1 (2.7%)	0.459	NA	NA
Adverse drug reaction	0 (0.0%)	1 (5.0%)	1 (2.7%)	1.000	NA	NA
Decreased platelet count	1 (5.9%)	0 (0.0%)	1 (2.7%)	0.459	NA	NA
Feeling cold	1 (5.9%)	0 (0.0%)	1 (2.7%)	0.459	NA	NA
Allergic cough	1 (5.9%)	0 (0.0%)	1 (2.7%)	0.459	NA	NA
Productive cough	0 (0.0%)	1 (5.0%)	1 (2.7%)	1.000	NA	NA
Cognitive disorder	1 (5.9%)	0 (0.0%)	1 (2.7%)	0.459	NA	NA

Apnoea, absence of respiratory flow for 10 seconds or more; chest pain, pain at the radiofrequency ablation thoracic site (visual analogue scale > 0); hypopnoea, 30% reduction in respiratory flow for 10 seconds or more; hypoxia, refers to hypoxaemia $SpO_2 < 90\%$; hypercapnia, $ETCO_2 > 5.3$kPa (40 mmHg); pain in extremity, positional arm pain (visual analogue scale > 0). Traumatic pneumothorax, clinical or radiological pneumothorax.

CI, confidence interval; CPAP+4, continuous positive airway pressure of 4 cm H_2O; NA, Not applicable; OR, odds ratio; Sham-CPAP, modified mask for placebo CPAP.

Supplemental Table 4.A. Maximum visual analogue scale values of arm positional pain and pain at radiofrequency site. Intention-to-treat population.

Variable	VAS	CPAP+4 (n=22) n (%)	Sham-CPAP (n=24) n (%)	Total (n=46) n (%)	*P*-value
Positional arm pain	VAS 0-3	10 (47.6%)	8 (33.3%)	18 (40.0%)	0.628
	VAS 4-6	4 (19.0%)	9 (37.5%)	13 (28.9%)	
	VAS 7-10	7 (33.3%)	7 (29.2%)	14 (31.1%)	
	Total	21 (100.0%)	24 (100.0%)	45 (100.0%)	
Pain at the RFA site	VAS 0-3	18 (81.8%)	16 (66.7%)	34 (73.9%)	0.339
	VAS 4-6	1 (4.5%)	5 (20.8%)	6 (13.0%)	
	VAS 7-10	3 (13.6%)	3 (12.5%)	6 (13.0%)	
	Total	22 (100.0%)	24 (100.0%)	46 (100.0%)	

Supplemental Table 4.B. Maximum visual analogue scale values of arm positional pain and pain at radiofrequency site. Per-protocol population.

Variable	VAS	CPAP+4 (n=17) n (%)	Sham-CPAP (n=20) n (%)	Total (n=37) n (%)	*P*-value
Positional arm pain	VAS 0-3	8 (50.0%)	6 (30.0%)	14 (38.9%)	0.443
	VAS 4-6	4 (25.0%)	9 (45.0%)	13 (36.1%)	
	VAS 7-10	4 (25.0%)	5 (25.0%)	9 (25.0%)	
	Total	16 (100.0%)	20 (100.0%)	36 (100.0%)	
Pain at the RFA site	VAS 0-3	14 (82.4%)	13 (65.0%)	27 (73.0%)	0.320
	VAS 4-6	1 (5.9%)	4 (20.0%)	5 (13.5%)	
	VAS 7-10	2 (11.8%)	3 (15.0%)	5 (13.5%)	
	Total	17 (100.0%)	20 (100.0%)	37 (100.0%)	

Results corresponding to the highest VAS value recorded at the cut-off points during the procedure.

CPAP+4, continuous positive airway pressure of 4 cm H_2O; RFA, radiofrequency ablation; Sham-CPAP, modified mask for placebo CPAP; VAS, visual analogue scale.

Supplemental Table 5.1.A. Tumour ablation margin. Intention-to-treat population.

Variable	Nodule	CT	Category	CPAP+4 (n=22) n (%)	Sham-CPAP (n=24) n (%)	Total (n=46) n (%)	*P*-value
Tumour ablation margin	Nodule 1	CT1	Complete	8 (42.1%)	8 (44.4%)	16 (43.2%)	1.000
			Incomplete	11 (57.9%)	10 (55.6%)	21 (56.8%)	
			Total	19 (100%)	18 (100%)	37 (100%)	
		CT2	Complete	10 (52.6%)	12 (66.7%)	22 (59.5%)	0.507
			Incomplete	9 (47.4%)	6 (33.3%)	15 (40.5%)	
			Total	19 (100%)	18 (100%)	37 (100%)	
		CT3	Complete	14 (70%)	15 (83.3%)	29 (76.3%)	0.453
			Incomplete	6 (30%)	3 (16.7%)	9 (23.7%)	
			Total	20 (100%)	18 (100%)	38 (100%)	
	Nodule 2	CT1	Complete	1 (50%)	0 (0%)	1 (25%)	1.000
			Incomplete	1 (50%)	2 (100%)	3 (75%)	
			Total	2 (100%)	2 (100%)	4 (100%)	
		CT2	Complete	2 (100%)	0 (0%)	2 (50%)	0.332
			Incomplete	0 (0%)	2 (100%)	2 (50%)	
			Total	2 (100%)	2 (100%)	4 (100%)	
		CT3	Complete	2 (100%)	0 (0%)	2 (50%)	0.334
			Incomplete	0 (0%)	2 (100%)	2 (50%)	
			Total	2 (100%)	2 (100%)	4 (100%)	
	Nodule 3	CT1	Complete	0 (0%)	0 (0%)	0 (0%)	NA
			Incomplete	0 (0%)	2 (100%)	2 (100%)	
			Total	0 (0%)	2 (100%)	2 (100%)	
		CT2	Complete	0 (0%)	0 (0%)	0 (0%)	NA
			Incomplete	0 (0%)	2 (100%)	2 (100%)	
			Total	0 (0%)	2 (100%)	2 (100%)	
		CT3	Complete	0 (0%)	0 (0.0%)	0 (0.0%)	NA
			Incomplete	0 (0%)	2 (100%)	2 (100%)	
			Total	0 (0%)	2 (100%)	2 (100%)	

Supplemental Table 5.1.B. Tumour ablation margin. Per-protocol population.

Variable	Nodule	CT	Category	CPAP+4 (n=17) n (%)	Sham-CPAP (n=20) n (%)	Total (n=37) n (%)	*P*-value
Tumour ablation margin	Nodule1	CT1	Complete	8 (50%)	8 (44.4%)	16 (47.1%)	1.000
			Incomplete	8 (50%)	10 (55.6%)	18 (52.9%)	
			Total	16 (100%)	18 (100%)	34 (100%)	
		CT2	Complete	10 (62.5%)	12 (66.7%)	22 (64.7%)	1.000
			Incomplete	6 (37.5%)	6 (33.3%)	12 (35.3%)	
			Total	16 (100%)	18 (100%)	34 (100%)	
		CT3	Complete	13 (76.5%)	15 (83.3%)	28 (80%)	0.690
			Incomplete	4 (23.5%)	3 (16.7%)	7 (20%)	
			Total	17 (100%)	18 (100%)	35 (100%)	
	Nodule 2	CT1	Complete	1 (50%)	0 (0%)	1 (25%)	1.000
			Incomplete	1 (50%)	2 (100%)	3 (75%)	
			Total	2 (100%)	2 (100%)	4 (100%)	
		CT2	Complete	2 (100%)	0 (0%)	2 (50%)	0.336
			Incomplete	0 (0%)	2 (100%)	2 (50%)	
			Total	2 (100%)	2 (100%)	4 (100%)	
		CT3	Complete	2 (100%)	0 (0%)	2 (50%)	0.332
			Incomplete	0 (0%)	2 (100%)	2 (50%)	
			Total	2 (100%)	2 (100%)	4 (100%)	
	Nodule 3	CT1	Complete	0 (0%)	0 (0.0%)	0 (0.0%)	NA
			Incomplete	0 (0%)	2 (100%)	2 (100%)	
			Total	0 (0%)	2 (100%)	2 (100%)	
		CT2	Complete	0 (0%)	0 (0.0%)	0 (0.0%)	NA
			Incomplete	0 (0%)	2 (100%)	2 (100%)	
			Total	0 (0%)	2 (100%)	2 (100%)	
		CT3	Complete	0 (0%)	0 (0%)	0 (0%)	NA
			Incomplete	0 (0%)	2 (100%)	2 (100%)	
			Total	0 (0%)	2 (100%)	2 (100%)	

Supplemental Table 5.2.A. Minimum halo thickness. Intention-to-treat population.

Variable	Nodule	CT	N CPAP+4	CPAP+4 n=22 Mean ± SD	N Sham-CPAP	Sham-CPAP n=24 Mean ± SD	Total n=46 Mean ± SD	*P*-value
Thickness (mm)	Nodule 1	CT1	19	2.16 ± 2.99	18	2.61 ± 3.29	2.38 ± 3.10	0.663
		CT2	19	2.84 ± 3.25	17	3.88 ± 3.37	3.33 ± 3.30	0.353
		CT3	20	4.35 ± 3.57	18	5.87 ± 3.34	5.08 ± 3.51	0.180
	Nodule 2	CT1	2	1.50 ± 2.12	2	0 ± 0	0.75 ± 1.50	0.500
		CT2	2	2.50 ± 0.71	2	0 ± 0	1.25 ± 1.50	0.125
		CT3	2	7.50 ± 4.95	2	0 ± 0	3.75 ± 5.19	0.278
	Nodule 3	CT1	0	-	2	0 ± 0	0 ± 0	NA
		CT2	0	-	2	0 ± 0	0 ± 0	NA
		CT3	0	-	2	0 ± 0	0 ± 0	NA

Supplementary Table 5.2.B. Minimum halo thickness. Per-protocol population.

Variable	Nodule	CT	N CPAP	CPAP+4 n=17 Mean ± SD	N Sham-CPAP	Sham-CPAP n=20 Mean ± SD	Total n=37 Mean ± SD	*P*-value
Thickness (mm)	Nodule 1	CT1	16	2.56 ± 3.10	18	2.61 ± 3.29	2.59 ± 3.15	0.960
		CT2	16	3.38 ± 3.28	17	3.88 ± 3.37	3.64 ± 3.52	0.664
		CT3	17	4.71 ± 3.50	18	5.89 ± 3.34	5.31 ± 3.42	0.313
	Nodule 2	CT1	2	1.50 ± 2.12	2	0 ± 0	0.75 ± 1.50	0.500
		CT2	2	2.50 ± 0.71	2	0 ± 0	1.25 ± 1.50	0.125
		CT3	2	7.50 ± 4.95	2	0 ± 0	3.75 ± 5.19	0.278
	Nodule 3	CT1	0	-	2	0 ± 0	0 ± 0	NA
		CT2	0	-	2	0 ± 0	0 ± 0	NA
		CT3	0	-	2	0 ± 0	0 ± 0	NA

Supplemental Table 5.3.A. Minimum halo thickness category. Intention-to-treat population.

Nodule	CT	Thickness category	CPAP+4 (n=22) n (%)	Sham-CPAP (n=24) n (%)	Total (n=46) n (%)	*P*-value
Nodule 1	CT1	< 5 mm	14 (73.7%)	12 (66.7%)	26 (70.3%)	0.727
		≥ 5 mm	5 (26.3%)	6 (33.3%)	11 (29.7%)	
		Total	19 (100%)	18 (100%)	37 (100%)	
	CT2	< 5 mm	14 (73.7%)	9 (52.9%)	23 (63.9%)	0.297
		≥ 5 mm	5 (26.3%)	8 (47.1%)	13 (36.1%)	
		Total	19 (100%)	17 (100%)	36 (100%)	
	CT3	< 5 mm	7 (35%)	4 (22.2%)	11 (28.9%)	0.485
		≥ 5 mm	13 (65%)	14 (77.8%)	27 (71.1%)	
		Total	20 (100%)	18 (100%)	38 (100%)	
Nodule 2	CT1	< 5 mm	2 (100%)	2 (100%)	4 (100%)	NA
		≥ 5 mm	0 (0%)	0 (0%)	0 (0%)	
		Total	2 (100%)	2 (100%)	4 (100%)	
	CT2	< 5 mm	2 (100%)	2 (100%)	4 (100%)	NA
		≥ 5 mm	0 (0%)	0 (0%)	0 (0%)	
		Total	2 (100%)	2 (100%)	4 (100%)	
	CT3	< 5 mm	1 (50%)	2 (100%)	3 (75%)	1.000
		≥ 5 mm	0 (0%)	0 (0%)	0 (0%)	
		Total	2 (100%)	2 (100%)	4 (100%)	
Nodule 3	CT1	< 5 mm	0 (0%)	2 (100%)	2 (100%)	NA
		≥ 5 mm	0 (0%)	0 (0%)	0 (0%)	
		Total	0 (0%)	2 (100%)	2 (100%)	
	CT2	< 5 mm	0 (0%)	2 (100%)	2 (100%)	NA
		≥ 5 mm	0 (0%)	0 (0%)	0 (0%)	
		Total	0 (0%)	2 (100%)	2 (100%)	
	CT3	< 5 mm	0 (0%)	2 (100%)	2 (100%)	NA
		≥ 5 mm	0 (0%)	0 (0%)	0 (0%)	
		Total	0 (0%)	2 (100%)	2 (100%)	

Supplemental Table 5.3.B. Minimum halo thickness category. Per-protocol population.

Nodule	CT	Thickness category	CPAP+4 (n=17) n (%)	Sham-CPAP (n=20) n (%)	Total (n=37) n (%)	*P*-value
Nodule 1	CT1	< 5 mm	11 (68.8%)	12 (66.7%)	23 (67.6%)	1.000
		≥ 5 mm	5 (31.3%)	6 (33.3%)	11 (32.4%)	
		Total	16 (100%)	18 (100%)	34 (100%)	
	CT2	< 5 mm	11 (68.8%)	9 (52.9%)	20 (60.6%)	0.481
		≥ 5 mm	5 (31.3%)	8 (47.1%)	13 (39.4%)	
		Total	16 (100%)	17 (100%)	33 (100%)	
	CT3	< 5 mm	5 (29.4%)	4 (22.2%)	9 (25.7%)	0.711
		≥ 5 mm	12 (70.6%)	14 (77.8%)	26 (74.3%)	
		Total	17 (100%)	18 (100%)	35 (100%)	
Nodule 2	CT1	< 5 mm	2 (100%)	2 (100%)	4 (100%)	NA
		≥ 5 mm	0 (0%)	0 (0%)	0 (0%)	
		Total	2 (100%)	2 (100%)	4 (100%)	
	CT2	< 5 mm	2 (100%)	2 (100%)	4 (100%)	NA
		≥ 5 mm	0 (0%)	0 (0%)	0 (0%)	
		Total	2 (100%)	2 (100%)	4 (100%)	
	CT3	< 5 mm	1 (50%)	2 (100%)	3 (75%)	1.000
		≥ 5 mm	1 (50%)	0 (0%)	1 (25%)	
		Total	2 (100%)	2 (100%)	4 (100%)	
Nodule 3	CT1	< 5 mm	0 (0%)	2 (100%)	2 (100%)	NA
		≥ 5 mm	0 (0%)	0 (0%)	0 (0%)	
		Total	0 (0%)	2 (100%)	2 (100%)	
	CT2	< 5 mm	0 (0%)	2 (100%)	2 (100%)	NA
		≥ 5 mm	0 (0%)	0 (0%)	0 (0%)	
		Total	0 (0%)	2 (100%)	2 (100%)	
	CT3	< 5 mm	0 (0%)	2 (100%)	2 (100%)	NA
		≥ 5 mm	0 (0%)	0 (0%)	0 (0%)	
		Total	0 (0%)	2 (100%)	2 (100%)	

Supplemental Table 5.4.A. Increase in tumour size from CT0 to CT3. Intention-to-treat population.

Variable	Nodule	N CPAP	CPAP+4 n=22 Mean ± SD	N Sham-CPAP	Sham-CPAP n=24 Mean ± SD	Total N=46 Mean ± SD	*P*-value
Increase in tumour size (mm)	Nodule 1	20	0.50 ± 3.85	18	1.28 ± 2.11	0.87 ± 3.13	0.439
	Nodule 2	2	-2.00 ± 1.41	2	2 ± 2.83	0 ± 2.94	0.215
	Nodule 3	0	-	2	5 ± 4.24	5 ± 4.24	NA

Supplemental Table 5.4.B. Increase in tumour size from CT0 to CT3. Per-protocol population.

Variable	Nodule	N CPAP	CPAP+4 n=17 Mean ± SD	N Sham-CPAP	Sham-CPAP n=20 Mean ± SD	Total N=37 Mean ± SD	*P*-value
Increase in tumour size (mm)	Nodule 1	17	0.06 ± 3.98	18	1.28 ± 2.11	0.69 ± 3.17	0.272
	Nodule 2	2	-2.00 ± 1.41	2	2.00 ± 2.3	0.00 ± 2.94	0.215
	Nodule 3	0	-	2	5.00 ± 4.24	5.00 ± 4.24	NA

Categorical variables are expressed as the number of cases (% of total cases) and continuous variables as mean ± SD. CPAP+4, continuous positive airway pressure of 4 cm H_2O; CT, computed tomography; CT1, computed tomography at the end of the procedure, electrode inserted, before stopping PSA. CT2, after removal of the ablation electrode and PSA, patient in supine decubitus, awake, CPAP mask removed; CT3: control CT scan at 24h prior to discharge. NA, not applicable; PSA, procedural sedation and analgesia; Sham-CPAP, modified mask for placebo CPAP.

A complete tumour ablation margin, a minimum halo thickness of 5 mm, and an increase in tumour size from TC0 to TC3, were indicative of the local efficacy of RFA.
